# Transcriptome analysis of the spider *Phonotimpus pennimani* reveals novel toxin transcripts

**DOI:** 10.1590/1678-9199-JVATITD-2022-0031

**Published:** 2023-01-23

**Authors:** Jonathan David Baza-Moreno, Leticia Vega-Alvarado, Guillermo Ibarra-Núñez, Karina Guillén-Navarro, Luz Verónica García-Fajardo, Verónica Jiménez-Jacinto, Elia Diego-García

**Affiliations:** 1 Grupo Académico de Biotecnología Ambiental, Departamento de Ciencias de la Sustentabilidad, El Colegio de la Frontera Sur (ECOSUR), Tapachula de Córdova y Ordoñez, Chiapas, Mexico.; 2 Instituto de Ciencias Aplicadas y Tecnología, Universidad Nacional Autónoma de México (UNAM), Ciudad de Mexico, Mexico.; 3 Grupo Académico de Ecología de Artrópodos y Manejo de Plagas, Departamento de Agricultura, Sociedad y Ambiente, El Colegio de la Frontera Sur (ECOSUR), Tapachula de Córdova y Ordoñez, Chiapas, Mexico.; 4 Instituto de Biotecnología, Universidad Nacional Autónoma de México (UNAM), Cuernavaca, Morelos, Mexico.; 5 Investigadora CONACyT - ECOSUR, Consejo Nacional de Ciencia y Técnología, Ciudad de Mexico, Mexico.

**Keywords:** Genes, Molecular diversity, Toxins, Transcripts, Wandering spider

## Abstract

**Background:**

*Phonotimpus pennimani* (Araneae, Phrurolithidae) is a small-sized (3-5 mm) spider endemic to the Tacaná volcano in Chiapas, Mexico, where it is found in soil litter of cloud forests and coffee plantations. Its venom composition has so far not been investigated, partly because it is not a species of medical significance. However, it does have an important impact on the arthropod populations of its natural habitat.

**Methods:**

Specimens were collected in Southeastern Mexico (Chiapas) and identified taxonomically by morphological characteristics. A partial sequence from the mitochondrial gene *coxI* was amplified. Sequencing on the Illumina platform of a transcriptome library constructed from 12 adult specimens revealed 25 toxin or toxin-like genes. Transcripts were validated (RT-qPCR) by assessing the differential expression of the toxin-like PpenTox1 transcript and normalising with housekeeping genes.

**Results:**

Analysis of the *coxI*-gene revealed a similarity to other species of the family Phrurolithidae. Transcriptome analysis also revealed similarity with venom components of species from the families Ctenidae, Lycosidae, and Sicariidae. Expression of the toxin-like PpenTox1 gene was different for each developmental stage (juvenile or adult) and also for both sexes (female or male). Additionally, a partial sequence was obtained for the toxin-like PpenTox1 from DNA.

**Conclusion:**

Data from the amplification of the mitochondrial *coxI* gene confirmed that *P. pennimani* belongs to the family Phrurolithidae. New genes and transcripts coding for venom components were identified.

## Background

Spiders (Order: Araneae) are members of the arachnids (Phylum: Arthropoda, Subphylum: Chelicerata, Class: Arachnida). With more than 50,200 described species distributed over 131 families [1], spiders are among the most abundant arthropod predators and controllers of insect pests in terrestrial ecosystems [2, 3, 4]. Spiders are divided into two large groups based on their predatory behavior, namely web-building and wandering spiders [5, 6]. Web-building spiders construct silken webs in which to capture prey (flying insects, for instance) [7, 8]. The families Araneidae, Deinopidae, Linyphiidae, Tetragnathidae, and Theridiidae (among others) can be found in this group [9]. Wandering spiders (such as the families Thomisidae, Lycosidae, Ctenidae and Phrurolithidae, among others) do not construct webs to capture their prey [10], but instead move about over the ground (including leaf litter) and vegetation in search of prey [5, 11, 12].

In 1940, Gertsch and Davis [13] described the then-new genus *Phonotimpus* and included two species, namely *Phonotimpus separatus* and *Phonotimpus eutypus*. In 2018, two new species from Southern Mexico (*Phonotimpus pennimani* and *Phonotimpus talquian*) were described by Chamé-Vázquez et al. [14]. The species *Phonotimpus marialuisae* (from the State of Mexico) was described in 2019 [15], and in 2021 the species *Phonotimpus padillai* was described [16] and the species *Gosiphurus schulzefenai* was transferred to the genus *Phonotimpus*. *Phonotimpus pennimani* is a wandering spider measuring less than 5 mm, and is commonly encountered in leaf litter on coffee plantations [14]. Angulo-Ordoñes et al. [6], studied the predatory behaviour of *P. pennimani* and its impact on other leaf litter inhabitants. Although indirectly, this species plays an important role in the recycling of nutrients in the local cloud forest ecosystem [6, 17].

Spiders produce venom in specialized organs with the goal of incapacitating their prey or defending themselves against predators [18, 19]. The molecular diversity of venom has been extensively explored in spider species of biomedical interest, but only limited information is available on genes, transcripts, or venom components of non-medically significant or small-sized species such as *P. pennimani*.

We aimed our attention on this little-studied endemic species to gain insight into the molecular composition of its venom. Spider venom is a mixture of various types of compounds, such as amino acids, enzymes, acylpolyamines, and peptides. Toxins are bioactive peptides that act with high affinity and specificity on various molecular targets, among them ion channels [20, 21]. These peptides can have a neurotoxic, cardiotoxic, antimicrobial, antifungal, or enzymatic action, can produce paralysis or death, and can aid prey digestion [22]. Considering the number of spider species and data from the mass spectrometric analysis of venoms, spiders may produce an estimated 2-20 million peptides [20, 23, 24].

The venom of wandering spiders of the genus *Cupiennius* (Trechaleidae) exhibits cytolytic activity and its components have been proposed as bioinsecticides [25]. The exploration of spider venoms has also brought about the development of bioinsecticides because of their selectivity for different targets such as sodium and calcium ion channels [23, 26]. The toxin omega-hexatoxin-Hv1a isolated from the wandering Australian funnel-web spider, *Hadronyche versuta* (Atracidae), exhibits high specificity and inhibits insect but not mammalian voltage-gated calcium channel (Ca_v_) currents [27]. Since it is a potent inhibitor of insect [28] Ca_v,_ omega-hexatoxin-Hv2a toxin has likewise been proposed as bioinsecticide [29]. Omega-theraphotoxin-Hg1a or SNX482 toxin from the African tarantula *Hysterocrates gigas* (Theraphosidae) has activity on R-type calcium ion (Ca_v_2.3) channels [30, 31].

Transcriptome analysis facilitates the identification of toxin-related sequences [9, 32]. The quantitative reverse transcription polymerase chain reaction (RT-qPCR), on the other hand, is a reliable detection and measurement technique for the quantification of gene expression, and is normalized to internal reference (housekeeping) genes.

This study aims to provide insight into the molecular diversity of the venom of the spider *P. pennimani*, a relatively underexplored species endemic to the Tacaná volcano in Chiapas, Mexico. A partial sequence from the mitochondrial cytochrome oxidase subunit I gene (*coxI*) allowed us to construct a molecular phylogenetic tree.

## Methods

### Collection of biological material

**Figure 1.**
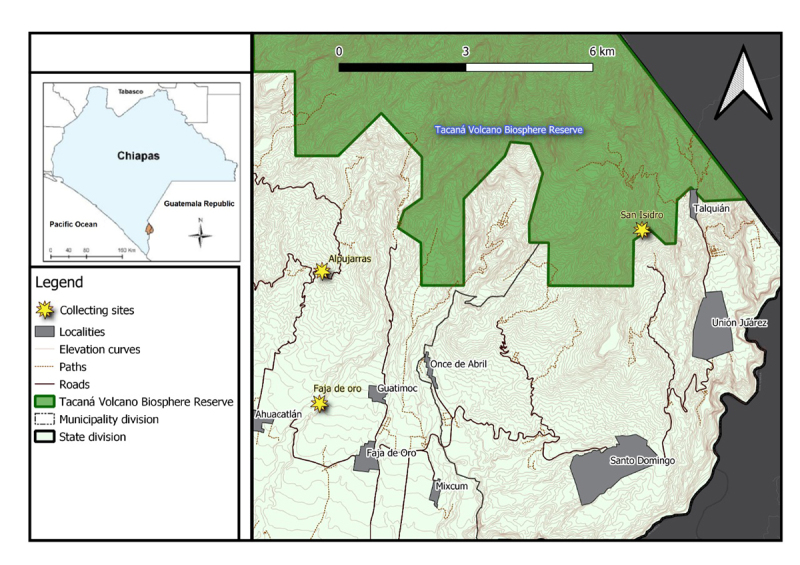
Map of Chiapas showing the *Phonotimpus pennimani* collecting sites. Yellow stars correspond to the collecting sites of Faja de Oro, Alpujarras (municipalities of Cacahoatán 14°59'29.49"N, 92°10'01.29"W; 986 m a.s.l.) and San Isidro (Unión Juárez 15°03'38.5"N, 92°04'54.77"W; 1701 m a.s.l.); the green area corresponds to the Tacaná biosphere reserve.

*Phonotimpus pennimani* specimens were collected in the communities of Alpujarras, Faja de Oro, and San Isidro, which are located in the municipalities of Cacahoatán (14°59'29.49"N, 92°10'01.29"W; 986 m a.s.l.) and Unión Juárez (15°03'38.5"N, 92°04'54.77"W; 1701 m a.s.l.) in the state of Chiapas, Mexico (Figure 1). The region is situated near the Tacaná volcano and its vegetation includes cloud forests and coffee plantations. Live specimens were collected from dry and humid leaf litter and transported to the laboratory. The identification of this small-sized (3-5 mm) spider was based on morphological characteristics according to the current taxonomic literature [14]. Following identification, specimens were preserved in RNALater (Sigma, USA) and stored at -20 ºC. 

### Nucleic acid extraction

Specimens were categorized by sex and developmental stage (juvenile and adult). Total DNA was extracted from the whole body (one to three specimens per extraction) using DNeasy Blood & Tissue kit (Qiagen, Germany) following the manufacturer's protocol. The extracted DNA was evaluated by gel electrophoresis (1% agarose gel) and quantified using a NanoDrop Lite spectrophotometer (Thermo Scientific, USA), after which the DNA samples were stored at -20 ºC.

RNA was extracted from the whole body of either males, females, or juveniles (one to 3 specimens per extraction) using an SV Total RNA Isolation System kit (Promega, USA) according to the manufacturer’s instructions. The extracted RNA was then visualized (1% agarose gel), quantified (NanoDrop Lite), and stored at -80 ºC until use in the RT-qPCR assay.

### Amplification of the coxI fragment

The *coxI* fragment was amplified by conventional polymerase chain reaction (PCR) using the oligonucleotide primer set LCO1490 and CHErev2. These primers have reportedly been used successfully for the amplification of the *coxI* fragment (around 720 pb) in invertebrates and Chelicerata [33, 34, 35, 36, 37]. PCR conditions were as follows: an initial denaturation step at 95 ºC for 3 min; followed by 32 cycles of 95°C for 1 min, 54°C for 1 min, and 72°C for 1 min; and a final extension for 10 min at 72 ºC.

PCR products were visualized on a 1.2 % agarose gel and the fragment was detected. PCR products were purified from agarose gel using the Wizard SV Gel and PCR Clean-up kit (Promega, USA) following the manufacturer’s instructions. Next, PCR products were visualized (1.2 % agarose gel) and quantified (NanoDrop Lite), and sent to Unidad de Síntesis y Secuenciamiento de DNA (USSDNA-UNAM; Cuernavaca, Mexico) for Sanger sequencing.

### Transcriptome generation

A *P. pennimani* transcriptome library was generated by using pooled total RNA isolated from twelve RNAlater-treated specimens (whole body; six males and six females). Libraries were prepared using RNAseq (Illumina, USA) and sequenced on the Illumina MiSeq platform (paired-end reads, 2 x 75) at the Unidad Universitaria de Secuenciación Masiva y Bioinformática (UUSMB-UNAM; Cuernavaca, Mexico).

### Amplification of *Phonotimpus pennimani* toxin-like 1 related fragments (PpenTox1)

The sequence TRINITY_DN123_c0_g1_i2 (named toxin-like 1 or PpenTox1) showed similarity to a reported spider toxin-like peptide (toxin CSTX-10, access number B3EWT0.2). Three primers were designed to amplify PpenTox1 (Table 1). DNA and cDNA were PCR amplified using the following program: an initial denaturation step at 95 ºC for 4 min; followed by 32 cycles of 95 ºC for 1 min, 50-58 ºC (gradient) for 1 min, and 72 ºC for 1 min; and a final extension for 10 min at 72 ºC. These primers target the complete toxin-like gene (381 bp), from the signal peptide to the stop codon (Met-stop fragment; primers 1 and 3) and including a Cys-rich region that possibly corresponds to the mature region (CIP-stop fragment, 201 bp; primers 2 and 3). Next, PCR products were cloned into a pJET plasmid vector using a CloneJET PCR Cloning kit (Thermo Scientific, USA), which was then used for the transformation of *Escherichia coli* DH5α cells. Colonies were screened for positive clones (those amplifying the desired PCR product) by Colony PCR. Plasmid DNA was isolated from positive transformants using the Column-Pure Plasmid Miniprep kit (Applied Biological Materials, Canada) as per the manufacturer’s instructions. Purified plasmids were sent to USSDNA-UNAM (Cuernavaca, Mexico) for Sanger sequencing.

**Table 1.** Primers designed for the amplification of PpenTox1, elongation factor-1-alpha, and succinate dehydrogenase from *Phonotimpus pennimani*.

Gene	Primer ID	Sequence	bp
Toxin-like PpenTox1	Primer 1 PpenTox1 Met	ATG AAA TTT CTA ACA ATT GAG GTT CTG	381
	Primer 3 PpenTox1 stop	TCA ACC GAC AAC ACT CTT TGC	
	Primer 2 PpenTox1 CIP	TGC ATT CCA AAG CAC CAC G	201
	Primer 3 PpenTox1 stop	TCA ACC GAC AAC ACT CTT TGC	
Elongation factor-1-alpha	EloFa For	GGA TGC TAT GGA ACC ACC TTC	218
	EloFa Rev	CAG GCA CAG CTT CCG TCA A	
Succinate dehydrogenase	SuccDe For	GCC ATC CAA CGA TTG GGA TTT G	137
	SuccDe Rev	CAG GCT GCT TAT AGC GTG TTT C

### Quantitative reverse transcriptase polymerase chain reaction

RT-qPCR was performed on total RNA obtained from one or two specimens of either females, males, or juveniles. cDNA was constructed from 50 ng RNA (25 μL final volume) using the Maxima First Strand cDNA Synthesis kit (Thermo Scientific, USA) following the manufacturer’s recommendations, and stored at -20 ºC until use.

Two specific primer pairs were designed based on the *P. pennimani* transcriptome sequences (Elongation factor-1-alpha (EloFa), sequences TRINITY_DN13_c0_g1_i2 and TRINITY_DN13_c0_g1_i1; Succinate dehydrogenase (SD), sequence TRINITY_DN8441_c0_g1_i2). Gene expression for the fragments of Table 1 was quantified by real-time PCR (1 μg cDNA, 0.125 μmol of each primer; 10 μL total volume) using the SsoAdvanced Universal SYBR Green Supermix kit (Bio-Rad, USA) as per manufacturer’s instructions. Each cDNA sample was analysed in triplicate for every primer pair. Three reactions without cDNA were included as control. RT-qPCR reactions were performed using a Bio-Rad CFX96 System (Bio-Rad, USA) and obtained products (10 μL) were visualised on 2% agarose gel.

Gene expression calculation was performed using BioRad CFX Maestro Software (version 2.3 v 5.3.022.1030). Normalized expression (DDCq) uses the calculated Relative Quantity (RQ):

=RQsample (GOI)RQsample (Ref 1) × RQsample (Ref 2) ×… × RQsample (Ref n)1n

Where RQ, Relative Quantity of a sample; Ref, Reference target in a run that includes one or more reference targets in each sample; GOI, Gene of interest (one target).

### Bioinformatics analysis

Sanger sequences were compared against the GenBank database with the BLAST algorithm (https://blast.ncbi.nlm.nih.gov/Blast.cgi) and multiple sequence alignments were generated using Clustal Omega (https://www.ebi.ac.uk/Tools/msa/clustalo/). Additionally, PpenTx1-related sequences were searched against the UniProtKB/Swiss-Prot database (https://www.uniprot.org/). The sequence alignment and generation of a 3D model of PpenTox1 with the purotoxin-2 template (PDB ID: 2MZF) was performed using the SWISS-MODEL server [38]. 

NGS transcriptome data quality was assessed using FastQC (version 0.11.9; https://www.bioinformatics.babraham.ac.uk/projects/fastqc/). Next, the software tool Trinity [39] was used to generate a de novo transcriptome assembly and the sequences were annotated using Trinotate (https://github.com/Trinotate/Trinotate.github.io/wiki/Loading-generated-results-into-a-Trinotate-SQLite-Database-and-Looking-the-Output-Annotation-Report). The program seqtk (https://github.com/lh3/seqtk) was then used to select only the sequences that matched specific filtering conditions. The transcriptome was translated into an amino acid sequence using the software tool Transdecoder (https://transdecoder.github.io). A BLAST search was performed against the UniProtKB/Swiss-Prot database to compare our data with previously reported sequences. Bioinformatics analysis data were plotted in the R programming language (version 4.0.5). Phonotimpus pennimani transcriptome sequences were deposited in the NCBI repository (Sequence Read Archive data or SRA). 

A phylogenetic tree was constructed for coxI sequences of P. pennimani and various Phrurolithidae sequences available in GenBank: Phrurotimpus alarius (HQ924602.1), Phrurotimpus borealis (JN308421.1), Phrurotimpus certus (KP649354.1), Scotinella pugnata (KT616693.1), Scotinella minnetonka (KP648502.1), Scotinella madisonia (MG047350.1), Scotinella fratrella (MG048225.1), Scotinella britcheri (JN308787.1), Liophrurillus flavitarsis (MW998035.1), Phrurolinillus tibialis (MW998464.1), Phrurolinillus lisboensis (MW998454.1), Phrurolithus szilyi (MW998512.1), Phrurolithus minimus (MW998482.1), Phrurolithus festivus (MW998474.1) and Phrurolithus nigrinus (MT607867.1). Two species from the genus Cithaeron were used as outgroup to root the tree, namely Cithaeron jocqueorum (KY017606.1) and Cithaeron praedonius (JQ412441.1). The phylogenetic tree was reconstructed in the MEGA X software program (Molecular Evolutionary Genetics Analysis) [40] using the maximum-likelihood method to estimate the tree of our data set (DNA sequences), nearest-neighbor-interchange (NNI) heuristic method and the Tamura-Nei model for multiple hits correction. The statistical robustness of the phylogenetic tree nodes was assessed by bootstrap resampling analysis (1000 replicates).

## Results

### Collection of nucleic acid and its extraction from Phonotimpus pennimani specimens

A total of 42 P. pennimani specimens (3-5 mm; 17 females, 8 males, and 17 juveniles) were collected from different sites and identified taxonomically. Specimens were collected from different sites between December 2019 and February 2021. The 12 specimens used for the generation of the transcriptome were collected between January and June 2020 in the locality of Alpujarras. Total DNA was obtained for each group separately (33.4 ng/μL and 17 ng/μL for females and males, respectively), but one experiment (DNA_Mix) included specimens from the two groups together. RNA extraction yielded 51, 45, and 70 ng/μL for females, males, and juveniles, respectively. This material was used for validation of expression by RT-qPCR. 

### Amplification of the coxI fragment

**Figure 2.**
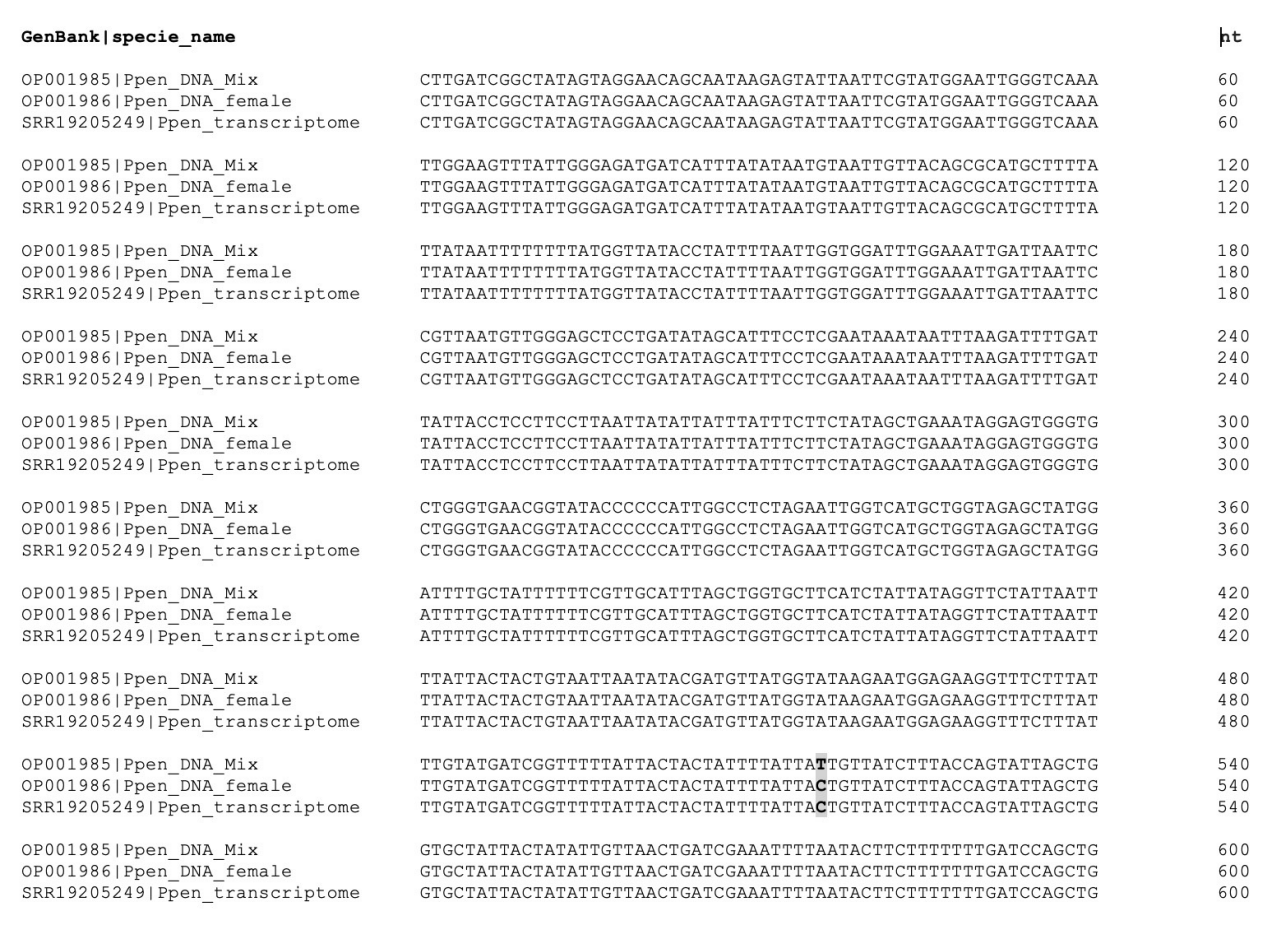
Multiple sequence alignment of Phonotimpus pennimani coxI fragment. Ppen: Phonotimpus pennimani; DNA_Mix corresponds to amplified sequence from total DNA of a mixed population (females and males; GenBank OP001985); female_DNA corresponds to sequences amplified from total DNA of a population of females (GenBank OP001986); transcriptome corresponds to a sequence obtained from the P. pennimani transcriptome (SRA: SRR19205249, ID sequence TRINITY_DN20655_c0_g1_i1).

**Figure 3.**
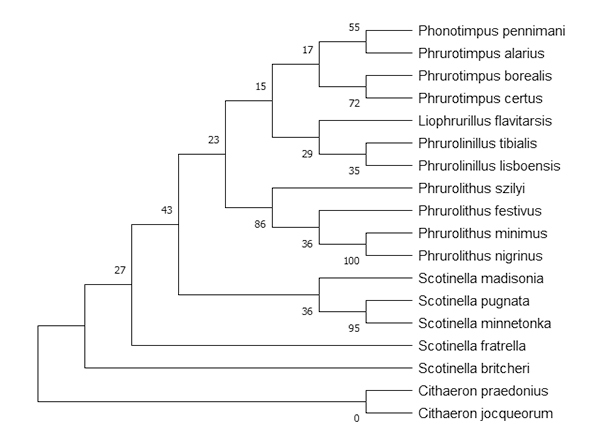
Phylogenetic tree of the family Phrurolithidae based on mitochondrial cytochrome oxidase subunit I (600 bp) nucleotide sequences that are available in databases and the coxI sequences generated in the present study (P. pennimani, Ppen_DNA_Mix GenBank OP001985; Ppen_DNA_female GenBank OP001986).

A coxI fragment was amplified by PCR using genomic DNA that was isolated from either only female specimens (DNA female) or from a mix (DNA_Mix). A region of 600 bp was selected for sequence analysis (sequenced from both ends). The DNA sequences are listed in Figure 2; the only nucleotide change (position 516, T/C) is highlighted. This nucleotide change is linked to the DNA source (female-only or mixed), which was verified by comparison with transcript TRINITY_DN20655. Two sequences (from multiple sequence alignment) were deposited in GenBank (Ppen_DNA_Mix OP001985; Ppen_DNA_female OP001986). The P. pennimani coxI fragment was then compared with data from other phrurolithid spider species (Additional file 1). It showed 90% similarity with Phrurotimpus alarius (HQ924602.1) and 88% with Scotinella pugnata (KT616693.1), which was also the closest relative to P. pennimani in the phrurolithid phylogenetic tree (Figure 3).

### Phonotimpus pennimani transcriptome

Phonotimpus pennimani transcriptome was deposited in the SRA of NCBI under BioProject PRJNA837986, with BioSample: SAMN28240954, and SRA: SRR19205249 (https://dataview.ncbi.nlm.nih.gov/object/PRJNA837986?reviewer=orl60agvh21lijl3lrv9nhci2). Sequencing of the transcriptome produced 14804989 raw reads. Sequence quality was verified using FastQC software (FastQC quality more than Q30). Data included the Q20, Q30, and GC content of the clean data. Sequence fractions with more than Q30 were considered high-quality reads. A total of 87800 transcripts with an average length of 341 bp (range: 55-7956 bp) were identified. These were assembled using Trinity and 79670 unigenes were obtained. A total of 27641 unigenes were annotated using Trinotate (databases used: sprot_Top_BLASTX_hit, RNAMMER, sprot_Top_BLASTP_hit, Pfam, SignalP, TmHMM, eggnog, Kegg, gene_ontology_blast, gene_ontology_pfam). Filtering for keywords of interest (possible venom compounds) revealed 26 matches for “Ctenidae” and “Phoneutria”, 11 for “Cupiennius”, 175 for “toxins”, and no matches for “Phonotimpus” or morphologically related family and genera (Phrurolithidae, Trachelidae, Drassinella, Otacilia). 

**Figure 4.**
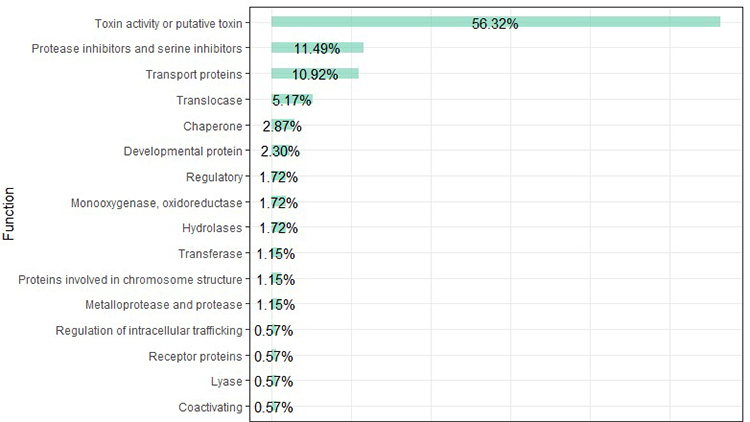
Classification of Phonotimpus pennimani transcripts according to their function (as reported in NCBI and UniProtKB).

**Figure 5.**
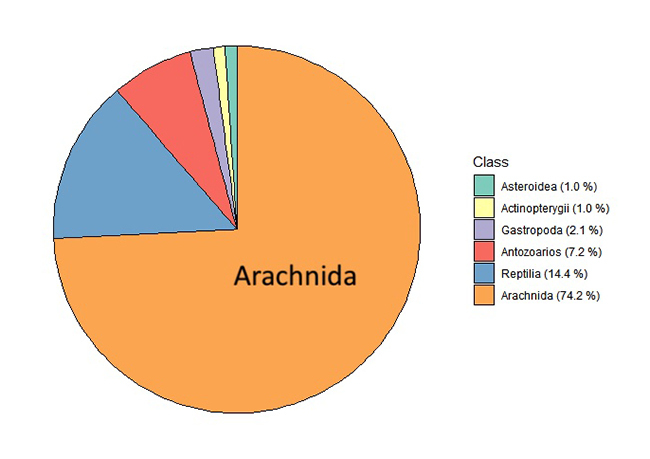
Similarity percentages (at class level) of Phonotimpus pennimani transcripts related to toxins or putative toxins (Figure 3; 56.32%).

Figure 4 shows the classification of 212 transcripts according to their function (as reported in NCBI and UniProtKB; Additional file 2). Fifty-six percent of the transcripts corresponded to toxins or putative toxins and were marked as venom components. The remaining 43% participate in a variety of other cellular activities. Seventy-four percent of the venom component transcripts showed similarity to toxins or venom components documented for Arachnida, whereas the remaining transcripts were related to venom components from a variety of vertebrates and invertebrates (Figure 5). Spider toxin-related transcripts showed similarity to venom components from families such as Sicariidae, Lycosidae, Agelenidae, Araneidae, and Ctenidae (Additional file 3). Some of the transcripts, however, showed similarity to putative toxins or venom compounds (such as enzymes or metalloproteinases).

### Toxin-like genes and PpenTox1

A total of 25 transcripts were identified that coded for toxins or toxin-like peptides similar to those described in the arachnid families Ctenidae, Lycosidae, Sicariidae, Ixodidae and Araneidae (Table 2). Twenty-three of them are related to spider venom components. Three transcripts moreover show identity with dermonecrotic spider toxins (TRINITY_DN70170_c0_g1_i1, TRINITY_DN2497_c0_g1_i1, and TRINITY_DN78624_c0_g1_i1). Two transcripts were determined to be similar to metalloproteases from Loxocelles intermedia, namely metalloprotease toxin1 (TRINITY_DN71263_c0_g1_i1) and metalloprotease toxin2 (TRINITY_DN37401_c0_g1_i1). Three sequences showed similarity to toxins from Phoneutria nigriventer and toxin-like peptide from Lycosa singoriensis (TRINITY_DN71_c0_g1_i2, TRINITY_DN71_c0_g1_i3 and TRINITY_DN1415_c2_g1_i1). Other sequences showed similarity to transcripts structurally related to venom components from Phoneutria nigriventer, Cupiennius salei, Caerostris extrusa and Agelena orientalis (Additional file 3).

Three P. pennimani sequences show similarity to spider dermonecrotic toxin. The largest sequence (TRINITY_DN2497_c0_g1_i1) shows 59% identity to dermonecrotic toxin StSicTox-betaIB1i-like from the African social spider Stegodyphus dumicola (GenBank XP_035213733.1), in a region with 329 aa (e-value: 7e-146). The sequences TRINITY_DN70170_c0_g1_i7 and TRINITY_DN 78624_c0_g1_i1 are shorter partial sequences related to the dermonecrotic toxins LiSicTox-betaID1 and LspiSicTox-betaIE respectively (Table 2).

**Table 2.** Amino acid sequences translated from Phonotimpus pennimani transcripts that showed similarity to toxins or other arachnid venom components.

*Transcript ID P. pennimani*	Translated amino acid sequence (precursor)	Similarity (UniprotKB)	Related toxins (Identity %)
TRINITY_DN70170_c0_g1_i1	ARGMESRLKDVGFDGGEDDLGETRRMFARLGIQSNVWQGDGKSNCLSVFYPDTRLRREVAIREASNGYIQKVYHWTIDLKLRMRLSLNIGVDGMITNDPEDLVEVLQESYFRNEFRLATPDDDPFTRFEGKC	A411_LOXHI	Dermonecrotic toxin, LhSicTox-alphaIV1i (45.80); SdSicTox-betaIIB1bxii-like (75)
TRINITY_DN2658_c0_g1_i1	STQILVLLLVAVLAAFVVPSLGVSCRTQPDMCGEIQCCVEFGMFGICRSLLKEGDKCELSSLKSKESDHVYLMKCPCGEGLTCKEDDSGSAIQGKCVAK	VP164_LYCMC	Venom protein 164 (35.87)
TRINITY_DN2497_c0_g1_i1	MNKTWRFLLCIIIYLFKITCAVEKILEFEEDIRRPIWNIAHMVNALYQVDYYLDRGANGLEFDISFDWDGNARYTYHGVPCDCFRSCLRYEYIDTYLDYLRKLTTPGHPQYRQQLVLLFMDVKVQQLSHEAKIRAGEDLATKLLDHYWKRGLSNGAANILVSVPSIRHMDFVDSFRGKLKSEGVADLYEIKIGYDFSGNEDLNNIQYTCQSNNLNAHIWQGDGITNCLPRGTGRLQEAINKRDRSAVEFIDKVYWWTVDKRSTMRKTLSMGIDGLITNYPDALISVLNEEEFSRKLRLATYEDNPWDHYANPTARLISDIQQAREEYNTTYQNDDELLYNC	B1Q_LOXIN	Dermonecrotic toxin LiSicTox-betaID1 (50.49)
TRINITY_DN71263_c0_g1_i1	ICYNLGKMDVSWIMFALSALSLNSYILGGNPMEMKELFEGDIAGIDPFVLLDRNAVVDEAELWPGGIVYYEIDWKLSHVKDVVLEAIDEYESKTCLKFRPKTASTKDYVKFTIVTGCWSSVGRKGGEQEISLSEGCHDKVSAVHELGHAVGLWHEHSRSDRDDYLEILWDNIKPGSEHNFLKLKPWENNLLGEEFDYKSIMLYGEYAFAKDRKSMTMRPRKDVVIGLINDKPGLSDSDVRRINKLYQCFGNERPPPPEVPDFKCDFEVDMCGLVNHENNQNAQWMVNKSELGGRTGSYLSVNAKDASFRRVRLVTPFFAAYGRKNGCFRFEVYFNGGGAVSLDVMMHSVKTSKLVLKHIDKMESWQPIALDVDLGGDIKFSLDARTRKSDGEGIIALDNIQYSLREC	VMPA_LOXIN	Metalloproteinase (47.25)
TRINITY_DN123_c0_g1_i1	MKFLTIEVLFASALFFTAICRCLTETGEDFKDALEEGSRNLQEDSGKIELNEEPRSSKCTKRNHDCTSDRHSCCRSKMFKDVCECFYKEGNDTAKAEEMCTCQQPMHLKMIEEGFQKAHDFVGR	TXZ01_LYCSI	Structure similar to toxin LSTX-D1 (46.28)
TRINITY_DN123_c0_g1_i2	MKFLTIEVLFASALFFTAICRCLTETGEDFKDALEEGSRNLQEDSGKIELNEEPRETEERCIPKHHECTHQKDNCCKSKGHVLPNKCHCYKVENEADEVTRRCACITPMQYKPIELAATFAKSVVG	TX501_LYCSI	U5-lycotoxin-Ls1a (36.51)
TRINITY_DN170_c0_g1_i3	DDSRLQFGKMKLLLCSVLLLLAVAACIEAQSNNKDCIKYENLCTDNTDSCCSGLICQCLERYHGTETGATKCWCIEHDILYRDSVKVPDQKSLLHPSGIMQSKKKQILGTV	TX606_LYCSI	U6-lycotoxin-Ls1f (42.86)
TRINITY_DN1415_c2_g1_i1	MILLSQSTNNSTQQKMKFLFVVLLLAVAVIYIEVEAEGSCVNYEEECTVDKSGCCSGLKCDCYSVTIKGTQAPDKCWCLEQDITYGALE	TX610_LYCSI	U6-lycotoxin-Ls1b (45.76)
TRINITY_DN547_c0_g1_i4	FFFTHLCGVMFNALKIGNERATKMEKIVSFFIFITSILCEVSGQKNIVRKMQKIVLFSFIAVIFIAVSAERGRYCPTKSNAICIHRWDQCCSYEDCPPRQLCCDEQCGNTCHPPVSEPTTGSRVDYDPNCRIGSG	TXE04_LYCSI	U14-lycotoxin-Ls1b (39.68)
TRINITY_DN547_c0_g1_i2	FFFTHLCGVMFNALKIGNERATKMEKIVSFFIFITSILCEVSGQRRYCPTKSNAICIHRWDQCCSYEDCPPRQLCCDEQCGNTCHPPVSEPTTGSRVDYDPNCRIGSG	TXAG7_AGEOR	U7-agatoxin-Ao1a (40.39)
TRINITY_DN5823_c0_g1_i4	RSESTVAVPTPSGKEGITVFPSSQFDTGKHQTKSFNFIGKHEIPVEAFGCNRSTIPMLCSHGQCLCNAWLFLIRQFSGQLPGTQLRSAPPVPDVNTEKELHPA	TXAG8_AGEOR	U8-agatoxin-Ao1a (31.92)
TRINITY_DN221_c0_g1_i1	MRTVIFVTLVCCISLVSAENEEKAKCQSNSDCGDGECCVNIHDYTESVCKKLRQKDDFCFPNDEWNVVGEGATYRYKYMCPCLDGLECKAAEVKEENGVTTYVGAKCGTFGKY	TXCA_CAEEX	U3-aranetoxin-Ce1a (37.08)
TRINITY_DN78624_c0_g1_i1	GSVREVFHGPPCDYLRNCTRRADLQEFLTYVRNITDPSFPGNYGQKMVMQFFDLKLG	B1R2_LOXSN	Dermonecrotic toxin LspiSicTox-betaIE1ii (39.29)
TRINITY_DN112_c0_g1_i3	MYIVYFRKLLDSLEAVIDDVCLLTPAFASYFNKVRTEISPVTPTRGSFTARGSIRRTYIFIRNTVCFNVIDQKYQKEGGHQ	TX711_LYCSI	U7-lycotoxin-Ls1c (42.31)
TRINITY_DN170_c0_g1_i4	DDSRLQFGKMKLLLCSVLLLLAVAACIEAQSNNKNCIRYENPCTDDRDNCCSGLPCRCFDRVDGDVMGTRKCWCLESDIGLKIVLSE	TX602_LYCSI	U6-lycotoxin-Ls1c (42.86)
TRINITY_DN170_c0_g1_i9	DDSRLQFGKMKLLLCSVLLLLAVAACIEAQSNNKNCIRLENVCTDDSNCCSGLACRCFDRSVDDADGARKCWCLESDIGLKIVLSE	TX830_LYCSI	U9-lycotoxin-Ls1a (45.07)
TRINITY_DN44322_c0_g1_i1	MLLINLAMAISCARYFALFMLFGSCVCDCRRPFYVIGHMVNSIEEIMPYLDRGANVIETDIQFHPNGS	SMD_IXOSC	Dermonecrotic toxin SPH (52.63)
TRINITY_DN1195_c0_g1_i1	MDFVKMFLVNLTLFLIALVVCVVSDEPGFCPGYTPRECPYKINDCCVQADCPSYAICCEQPCGNVCRHKAARAIGTPLKDGTECKLGRVDPKRWYEKLFG	TXK04_LYCSI	U20-lycotoxin-Ls1d (43.68)
TRINITY_DN1660_c0_g1_i1	SDCIDISVTAQILKTTMKTITTMVALLLVTLVVVAATQMVDAEEIQEQERGFCAQKGIKCNDIHCCTGLKCSCAGSKCVCKPK	TXAG4_AGEOR	U4-agatoxin-Ao1a (57.45)
TRINITY_DN37401_c0_g1_i1	VKDKGCRAVVGYIGRRQRLTLGTGCIWVARVLHELFHVLGFFHEHTRPDRDEYVTVYEDNIKAASLNNFR	VMPA2_LOXIN	Toxin 2 of metalloproteinase (53.62)
TRINITY_DN36352_c0_g1_i1	FASELVKRNKEIMRSTLILTVLAVIAVSAVYARPQSDCEKHRESAEKMETIMKLIPKCKENGDYEELQQYKDSDFKVCYDKKGHPVSPISSKLTECNCHLKRKQKMDLNLGPDAYIPQCEEDGKWAKKQIWDYNGSCWCVDEKGETVGKVTHADNCKTLRCE	PN16_PHONI	U24-ctenitoxin-Pn1a (69.47)
TRINITY_DN71_c0_g1_i2	MKMLSKKFLYVFATVLISLIAARAEPEEAENEVAPEERAGKCIKAYKYGCRYPEKPCCEGTNCVCSFAMTNCQCKLPIGKVVKELFGFSK	TX31_PHONI	Kappa-ctenitoxin-Pn1a (41.67)
TRINITY_DN71_c0_g1_i3	MKMLSKKFLYVFATVLISLIAARAEPEEAGNEVAPEEARGCLEVYAHGCHYPEKPCCGGRTCKCSIAMTNCQCKKTLGELFGFSK	TX3A_PHONI	U6-ctenitoxin-Pn1a (49.38)
TRINITY_DN1379_c0_g1_i1	LFTVCFIMKVTFGLLVLCGVVAISIACENQSDCAEDECCTFDFKDDPHCEKRYGAGEKCPDTVLYAEHSDTFLMGCPCVQGYECLGRRVTVNGKTVKNTTCIMPL	TXC20_CUPSA	Toxin CSTX-20 (52.38)
TRINITY_DN55842_c0_g1_i1	MAIKYLTLLCSFCLYTSTCVSSFQDITIPNCGRSILSTASPDRIVGGKDAKHGQYPWMVSLQENADSVFEHVCGAAILNEYWIVTAAHCIELINQPWKYQVLVGLNKLSEQNAPTVQRISISKIIINDNYNDEDFRNDIALLKMAKPIDFAGSNGYVNGICLPETNNDPTGYAIVTGWGHTYEDGRNSNILKEVVVPVIPRDVCNKAYDDDPFDGLDEVTESMLCAGMASRDSCQNDSGGPLIQKSSDGRAILIGIVSNGTGCGDRNYPGIYTKISSYKEWIRNTMENYK	PN47_PHONI	U21-ctenitoxin-Pn1a (46.94)

**Figure 6.**
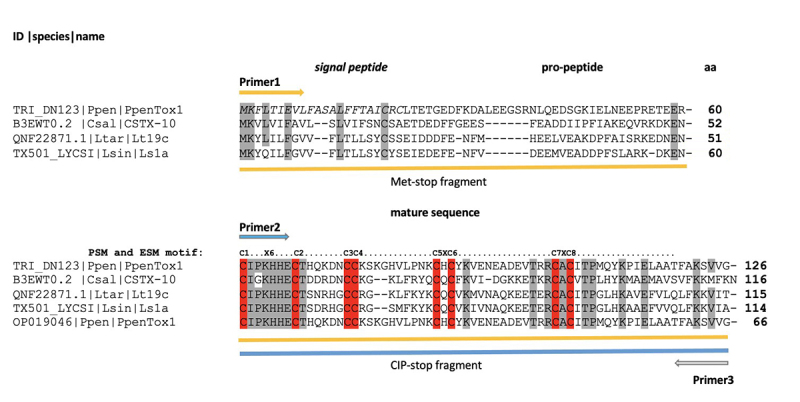
Multiple sequence alignment of amino acid sequences translated from the transcript sequence TRINITY_DN123_c0_gl_i2 (toxin-like PpenTox1). Ppen: Phonotimpus pennimani; Csal: Cupiennius salei; Ltar: Lycosa tarantula; CSTX-10: C. salei toxin CSTX-10 (GenBank B3EWT0.2); Lt19c: L. tarantula toxin U2-lycotoxin-Lt19c (GenBank QNF22871.1); Ls1a: L. singoriensis toxin U5-lycotoxin-Ls1a (UniProtKB B6DCV0). The underscored part corresponds to the Met-stop fragment (381 bp; primers 1 and 3) and the Cys-rich truncated CIP-stop fragment (201 bp; primers 2 and 3; GenBank OP019046), which was used in PCR amplification from DNA and qPCR experiments. The Cys residues that participate in folding are indicated in red. The PSM and ESM Cys distribution patterns are shown in the horizontal bar above the CIP-stop sequences. Arrows correspond to the primers from Table 1.

**Figure 7.**
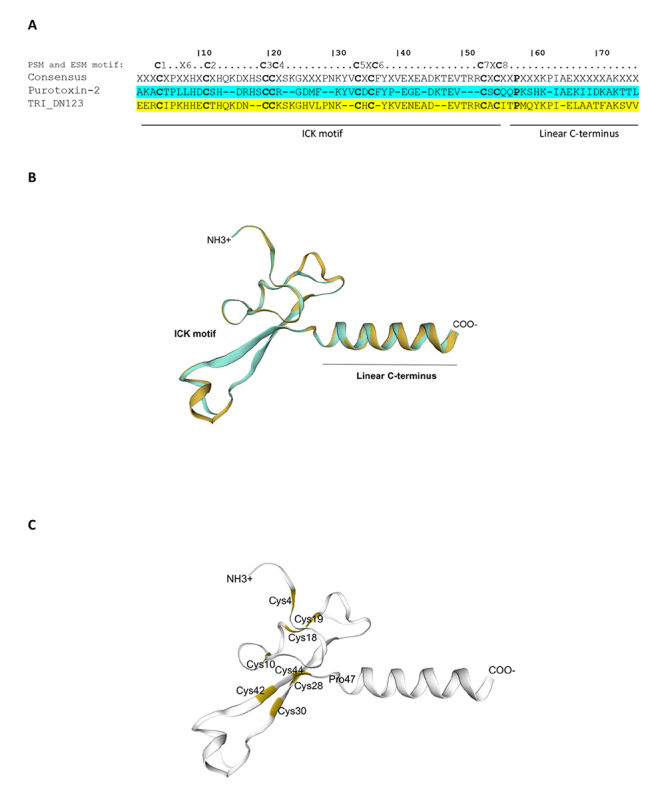
Sequence alignment of toxin-like PpenTox1 and 3D model. **(A)** PpenTox1 sequence (transcript TRINITY_DN123_c0_gl_i2) was aligned with purotoxin-2 as template (PDB ID: ID:2MZF). **(B, C)** Three-dimensional model was generated using SWISS-MODEL. The color aqua green represents purotoxin-2, while yellow corresponds to PpenTox1. Cys residues are in bold font (panel **A**) and are indicated in panel **C**. Cys-rich ICK motif and linear C-terminal are indicated in the alignment and 3D model.

Evaluation of amino acid sequences derived from the transcriptome indicated the presence of Cys-rich structures and toxin-like peptides (Additional file 3). Transcript sequence TRINITY_DN123_c0_g1_i2 was identified as a toxin-like peptide and named PpenTox1 (Figure 6). This sequence shows similarity to CSTX-10, a toxin with calcium channel blocking activity found in the American wandering spider Cupiennius salei. The NMR structure of purotoxin-2 (a toxin from the Wolf spider Alopecosa marikovskyi) was used as template to generate a 3D structure model for the toxin-like PpenTox1 (Figure 7) [41]. The purotoxin-2 structure (PT2; UniProt B3EWH0, PDB ID: 2MZF) contains an ICK (or “knottin”) motif in the N-terminal region) and a C-terminal linear cationic domain.

PpenTox1 was chosen for the design of specific primers for the validation of expression by RT-qPCR, and to obtain a partial sequence of the gene from the total DNA. Two PCR products were recovered: one product corresponded to the complete PpenTox1 gene (Met-stop fragment), whereas the second represented a Cys-rich truncated gene (CIP-stop fragment; Figure 6). The CIP-stop fragment was Sanger sequenced and compared with transcriptome data to confirm the identity of the product.

### Validation of differential expression by RT-qPCR

Examined RNA samples were derived from 1-3 juvenile or adult specimens (female and male adults were treated separately). The PpenTox1 transcript was stable, and the EloFa and SD genes were normalized (Additional file 4). 

Although most RT-qPCR primers are designed to render smaller amplicons, we aimed to validate the presence of either complete or truncated PpenTox1 gene expression. Primers were validated using melting curves to ensure a single PCR product. After each RT-qPCR run, amplicons were visualized in 2% agarose gels (Additional file 5). Then, 10 µL PCR amplicon was loaded in 2% agarose gel. This information is provided as supplementary material (Additional files 4 and 5).

**Figure 8.**
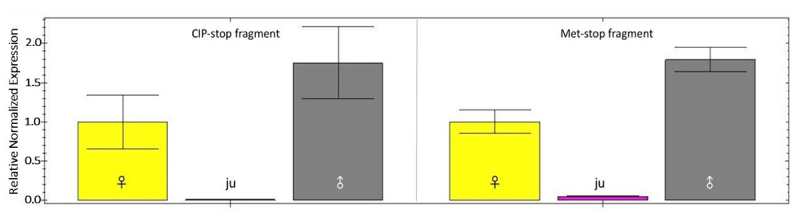
Expression of the PpenTox1 gene in a population of male, female, or juvenile Phonotimpus pennimani spiders, normalized with the reference genes EloFa and SD. CIP-stop fragment: expression of the truncated region of PpenTox1 (CIP-stop, 201 bp); Met-stop fragment: expression of the complete PpenTox1 transcript (Met-stop, 381 bp); ju: expression in a population of juveniles; ♂: expression in a population of males; ♀: expression in a population of females. The relative quantification (normalized to the reference genes) shows the standard deviation from a number of repetitions per sample.

The results from the validation of expression indicate that the expression of the toxin-like PpenTox1 gene was different for each developmental stage (juvenile vs. adult) and also for both sexes (male vs. female) (Figure 8). This difference is observed in the expression of both PpenTox1 fragments (Met-stop and CIP-stop).

## Discussion

The coxI fragment of P. pennimani was amplified by using the previously reported primers set LCO1490/CHErev2 [32, 33]. Vidergar et al. [37] used these primers to amplify the coxI gene in various species of wandering spiders, such as Clubiona terrestris, Evarcha arcuata, and Misumena vatia. The limited available sequence information about the spider family Phrurolithidae concentrates on species from the genera Scotinella, Otacilia, Liophrurillus, Phrurolinillus, Phrurolithus, and Phrurotimpus. The P. pennimani coxI sequences from the present study showed 87% and 89% similarity with Scotinella and Phrurotimpus, respectively, which indicates that they belong to the same family. Our results support the phylogeny proposed by Penniman [42], who already pointed out the close relationship between the genera Phonotimpus, Piabuna, Scotinella, and Phrurotimpus (based on morphology). The close relation between Phonotimpus, Scotinella, and Phrurotimpus was substantiated by the construction of a phylogenetic tree based on coxI sequences available in databases and the coxI sequences generated in our study (Figure 3). Our phylogenetic analysis considers a single gene as molecular marker.

In a new phylogeny proposal based on the revision of the morphological character system of dionychan spiders, Ramírez [43] concluded that Trachelidae is one of the families closest to Phrurolithidae. Only one transcriptome-based phylogenetic study of trachelid spiders has so far been reported [44]. In that publication, the transcriptome of Trachelas tranquillus (BioProject: PRJNA251570, SRA: SRX567376) was reported although it was not the focus of the study. Thus far, no transcriptome studies on phrurolithid spiders have been published, which makes our study the first to present transcriptome data of a phrurolithid species. 

Spider venom is composed of a variety of compounds, including toxins [45]. Toxins are peptides that often act selectively on a particular molecular target. Many spider toxins are ion-channel modulators that can modify ion channel gating. The calcium, potassium, and sodium ion channels attract the most medical attention because of their relation with several human diseases or disorders, such as heart arrhythmia, chronic pain, convulsions. We identified 23 transcripts in P. pennimani that coded for putative spider toxins. PpenTox1 showed 38% similarity with CSTX-10, a toxin that has been found in the wandering spider C. salei (B3EWT0.2). This spider is distributed over Mexico, Central America, and Hispaniola (World Spider Catalog, accessed May 2022) [1]. Kuhn-Nentwig [46] and co-workers first identified CSTX-10 in the venom of C. salei in 1994. It comprises 28% of the venom content [47], and inhibits L-type calcium ion channels (Ca_v_1/CACNA1) [48]. These channels are found in many cell types and are involved in brain development, heart cell function, and neurons. CSTX-10 toxin blocks Ca_v_1/CACNA1 in mammalian neurons and produces high voltage-activated calcium channels in cockroach neurons. PpenTox1 also showed 36% identity with the putative toxin U2-lycotoxin-Lt19c from the spider Lycosa tarantula (Figure 6). 

Some of the toxin-like coding transcripts showed similarity to the toxins Kappa-ctenitoxin-Pn1a (Tx31_PHONI) and U6-ctenitoxin-Pn1a (Tx3A_PHONI or neurotoxin Pn3) from the spider Phoneutria nigriventer (Table 2; Additional file 3). Kappa-ctenitoxin-Pn1a exhibits activity on Ca_v_1/CACNA1 channels [49]. Some transcripts show similarity with Loxosceles dermonecrotic toxins: TRINITY_DN70170_c0_g1_i1 (45 %, with LhSicTox-alphaIV1i), TRINITY_DN2497_c0_g1_i1 (50 %, LiSicTox-betaID1), and TRINITY_DN78624_c0_g1_i1 (57 %, LspiSicTox-betaIE1ii). These toxins induce hemolysis, massive inflammatory response (in mammals), and act on sphingomyelin [50, 51] (Additional file 3). Dermonecrotic toxin LiSicTox-betaID1 belongs to phospholipase-D family, the recombinant toxin (LiRecDT1) showed effect on Ca_v_1/CACNA1 and hemolysis of human erythrocytes [52].

Some putative toxins identified in the P. pennimani transcriptome contain a Cys-rich region that corresponds to the inhibitor cystine knot motif (ICK motif). The latter has been widely reported for various spider toxins and is characterized by three or four disulfide bridges (six or eight cysteines) [53]. Vassilevski et al. [53] suggested Cys (C) distribution patterns for the ICK motif. Two structural motifs with eight Cys can be distinguished in peptides, namely the principal structural motif (PSM) and the extra structural motif (ESM). PSM follows a C^1^X_6_C^2^…C^3^C^4^ pattern (X represents any amino acid residue), whereas ESM is characterised by a C^5^XC^6^…C^7^XC^8^ pattern [53]. The predicted structure of the mature peptide PpenTox1 includes eight Cys (C1 to C8) that are possibly involved in the formation of disulfide bridges and the ICK fold because PpenTox1’s Cys distribution pattern matches PSM and ESM (Figure 5). Also, 3D modelling and sequence alignment of purotoxin-2 (PT2) and PpenTox1 showed a significant alignment in the model (template PDB ID: 2MZF). Figure 7 shows the overlapping structures of PT2 and PpenTox1, and highlights the N-terminal region of the ICK motif and a C-terminal linear cationic domain. The toxins of this family target different membrane receptors. PT2 interacts with calcium channel and its linear domain is attributed antimicrobial properties [41]. The ICK motif, moreover, exhibits a rigid core that is stabilized by four disulfide bridges.

Transcriptome characterization can reveal gene expression profiles under specific conditions. Our report presents the first documented genes derived from the venom of a Phonotimpus spider. Gene expression data were validated by RT-qPCR. The reference genes EloFa and SD have previously been used in the validation of gene expression in other arachnids, for example in Panonychus citri [54]. The primers used for P. citri did not amplify the corresponding PCR product in P. pennimani (data not shown). Yet, we believe that the genes reported by Niu and co-workers are suitable because they express the genes across multiple developmental stages under abiotic stress. Transcriptome data of an adult population (males and females) was validated (RT-qPCR) by assessing the differential expression of the PpenTox1 transcript and normalizing using two genes (the EloFa and SD genes). The use of these reference genes significantly influenced observed differences. Our results coincide with Corzo and Escoubas [55], who demonstrated using mass spectrometry that arachnid venom composition varies between different species and between sexes of the same species. Especially in the cases of the tarantula Macrothele gigas [55] and Phoneutria boliviensis [56], there is a marked difference in venom composition between both sexes. 

EloFa and SD genes were selected based on gene expression studies in spider mites that tested several housekeeping genes in various developmental stages [54]. According to the software analysis of candidate reference genes (GeNorm, NormFinder, and Bestkeeper), these two genes proved more stable under stress conditions. We submitted our qPCR data to the same software analysis and to the comparative Delta-Ct method through the web-based tool RefFinder [57]. Results from the four software analyses are shown in supplementary material (Additional files 4 and 5). Lower ranking values correspond to more stable genes (Recommended Comprehensive Ranking). Based on the results (GeoMean Values: SD=1.19 and EloFa=1.41), we considered the selected housekeeping genes sufficiently stable for our purposes. Also, Genorm calculations indicated that by combining both genes, the stability value becomes 0.713, which is even less than the Geomean of all ranking values (Additional file 5). These data support the normalization of the target gene PpenTox1 with both housekeeping genes. 

Our study searched for similarities between P. pennimani transcripts and previously reported spider toxins. The generated data constitute the first report on the presence of toxins and venom component in this small leaf litter-inhabiting and predatory wandering spider. Nevertheless, a more exhaustive transcriptome analysis is required in which the different developmental stages and sexes are treated separately. This would allow the comparison of gene expression and the incorporation of proteomics data in the research of this relatively little-studied, yet ecologically important endemic species.

## Conclusion

Amplification of the mitochondrial coxI gene of P. pennimani advances the characterization of this species, complements the morphological phylogeny of the genus Phonotimpus in the family Phrurolithidae, and serves as a reference for future molecular phylogenetic analyses of this family. The transcriptome generated in our investigation provides some insight into gene expression in an adult P. pennimani population. The present study identifies the first phrurolithid venom-related transcripts and toxin-like peptides, and compares them with previously reported toxins from other arachnids.

### Abbreviations

Ca_v_1/CACNA1: L-type calcium ion channels; cDNA: complementary deoxyribonucleic acid; coxI: cytochrome oxidase subunit I mitochondrial gene; DNA: deoxyribonucleic acid; EloFa: elongation factor-1-alpha; ESTs: expressed sequence tags; MEGA-X: Molecular Evolutionary Genetics Analysis; NCBI: National Center for Biotechnology Information; NGS: next-generation sequencing; NNI: nearest-neighbour interchange; PpenTox1: Phonotimpus pennimani toxin-like 1; RNA: ribonucleic acid; RT-qPCR: real-time quantitative polymerase chain reaction; SD: succinate dehydrogenase. 

## Supplementary material

The following online material is available for this article:

Additional file 1. Multiple sequence aligment of the coxl fragment for species from the family Phrurolithidae. Abbreviations: Ppen_DNA, Phonotimpus pennimani (GenBank sequence OP001985); Pala_DNA, Phrurotimpus alarius (corresponding to fragment 24-623 nt of sequence HQ924602.1); Spug_DNA, Scotinella pugnata (corresponding to fragment 24-623 nt of sequence KT616693.1).

Additional file 2. Trinotate annotation of 212 Phonotimpus pennimani transcripts and their correspondence to Blastx and Blastp data after keyword filtering.

Additional file 3. Toxins and toxin-like peptides from Arachnida that show similarity to amino acid sequences derived from the Phonotimpus pennimani transcriptome.

Additional file 4. Elongation factor-1 alpha (EloFa) and succinate dehydrogenase (SD) have been used as RT-qPCR reference in various developmental stages of spider mites [54]. According to software analysis of candidate reference genes (GeNorm, NormFinder, and Bestkeeper). EloFa and SD genes are stable under stress conditions. We submitted our qPCR data to the same software analysis and also to the comparative delta-Ct method through the web-based tool RefFinder [57]. Results from the four software analyses are shown in **(A)** ranking order, better-good-average; and **(B)** comprehensive ranking, Geomean values: SD or SuccD = 1.19 and EloFa = 1.41, considering that lower ranking values indicate higher gene stability.

Additional file 5. Primers were validated through melting curves to ensure a single PCR product. After each RT-PCR run, amplicons were visualized using 2% agarose gels, as can be seen in the following images. About 10 μL PCR amplicon was loaded in 2% agarose gel. **(A)** ICK motif that corresponds to CIP-stop fragment; **(B)** complete PpenTox1 or Met-stop fragment; **(C)** and **(D)** elongation factor-1-alpha and succinate dehydrogenase fragments. L: 50 bp DNA ladder; Ju: population of juveniles; F: females; M: males; and NTC: not template curves.
