# Supplementary material for: Transcriptome analysis of the spider Phonotimpus pennimani reveals novel toxin transcripts
Source: J Venom Anim Toxins Incl Trop Dis. 2023 Jan 23;29:e20220031. doi: 10.1590/1678-9199-JVATITD-2022-0031 (PMC9881743; doi:10.1590/1678-9199-JVATITD-2022-0031)
Supplement: Additional file 1. [file 1678-9199-jvatitd-29-e20220031-s1.pdf]

J Venom Anim Toxins incl Trop Dis
https://doi.org/10.1590/1678-9199-JVATITD-2022-0031

Supplementary Material to "Transcriptome analysis of the spider *Phonotimpus pennimani* reveals novel toxin transcripts"

Additional file 1. Multiple sequence aligment of the *coxI* fragment for species from the family Phrurolithidae. Abbreviations: Ppen_DNA, *Phonotimpus pennimani* (GenBank sequence OP001985); Pala_DNA, *Phrurotimpus alarius* (corresponding to fragment 24-623 nt of sequence HQ924602.1); Spug_DNA, *Scotinella pugnata* (corresponding to fragment 24-623 nt of sequence KT616693.1).

```
GenBank|ID species
OP001985|Ppen_DNA          CTTGATCGGCTATAGTAGGAACAGCAATAAGAGTATTAATTCGTATGGAATTGGGTCAAA  60
HQ924602.1|Pala_DNA        CATGATCAGCAATAGTTGGAACTGCTATAAGAGTAATTATTCGAATAGAATTGGGTCAAG  60
KT616693.1|Spug_DNA        CGTGATCAGCTATAGTAGGAACAGCTATAAGAGTTATTATTCGTATAGAATTGGGTCAAG  60

OP001985|Ppen_DNA          TTGGAAGTTTATTGGGAGATGATCATTTATATAATGTAATTGTTACAGCGCATGCTTTTA  120
HQ924602.1|Pala_DNA        TTGGAAGATTATTAGGTGATGATCATTTATATAATGTGATTGTAACAGCACATGCATTTA  120
KT616693.1|Spug_DNA        TTGGAAGATTGTTAGGAGATGATCATTTATATAATGTAATTGTAACGGCACATGCATTTA  120

OP001985|Ppen_DNA          TTATAATTTTTTTTATGGTTATACCTATTTTAATTGGTGGATTTGGAAATTGATTAATTC  180
HQ924602.1|Pala_DNA        TTATAATTTTTTTTATAGTTATACCTATTTTAATTGGTGGATTTGGTAATTGGTTGATTC  180
KT616693.1|Spug_DNA        TTATAATTTTTTTTATAGTAATACCTATTTTGATTGGAGGCTTTGGTAATTGATTAATTC  180

OP001985|Ppen_DNA          CGTTAATGTTGGGAGCTCCTGATATAGCATTTCCTCGAATAAATAATTTAAGATTTTGAT  240
HQ924602.1|Pala_DNA        CTTTAATATTAGGAGCTCCGGATATAGCATTTCCTCGAATAAATAATTTAAGATTTTGAT  240
KT616693.1|Spug_DNA        CTTTAATATTAGGAGCGCCAGATATAGCCTTTCCTCGTATAAATAATTTAAGATTTTGAT  240

OP001985|Ppen_DNA          TATTACCTCCTTCCTTAATTATATTATTTATTTCTTCTATAGCTGAAATAGGAGTGGGTG  300
HQ924602.1|Pala_DNA        TATTACCTCCTTCATTAATTTTATTATTTGTTTCTTCTATAGCTGAAATAGGAGTTGGAG  300
KT616693.1|Spug_DNA        TGTTACCTCCTTCATTAATTTTGTTATTTGTATCATCTATAGCTGAAATAGGAGTGGGAG  300

OP001985|Ppen_DNA          CTGGGTGAACGGTATACCCCCCATTGGCCTCTAGAATTGGTCATGCTGGTAGAGCTATGG  360
HQ924602.1|Pala_DNA        CTGGGTGAACTGTATATCCTCCTTTAGCTTCTAGAATTGGACATTCTGGTAGTGCTATAG  360
KT616693.1|Spug_DNA        CTGGATGAACTGTATATCCTCCTTTAGCTTCTAGAGTAGGTCATTCTGGTAGAGCTATAG  360


OP001985|Ppen_DNA          ATTTTGCTATTTTTTCGTTGCATTTAGCTGGTGCTTCATCTATTATAGGTTCTATTAATT  420
HQ924602.1|Pala_DNA        ATTTTGCTATTTTTTCGTTACATTTAGCTGGTGCTTCTTCAATTATAGGTTCTATTAATT  420
KT616693.1|Spug_DNA        ATTTTGCTATTTTTTCTCTTCATTTAGCTGGTGCTTCTTCTATTATAGGATCTATTAATT  420

OP001985|Ppen_DNA          TTATTACTACTGTAATTAATATACGATGTTATGGTATAAGAATGGAGAAGGTTTCTTTAT  480
HQ924602.1|Pala_DNA        TTATTTCTACGGTTATTAATATACGTTCTTATGGAATAAGAATAGAGAAAGTACCTTTAT  480
KT616693.1|Spug_DNA        TTATTTCAACTATTATTAATATACGATCTTATGGAATAAGAATGGAAAAAGTTCCATTAT  480

OP001985|Ppen_DNA          TTGTATGATCGGTTTTTATTACTACTATTTTATTATTGTTATCTTTACCAGTATTAGCTG  540
HQ924602.1|Pala_DNA        TTGTATGATCAGTTTTTATTACAACTATTTTATTATTATTATCTTTACCAGTTTTAGCAG  540
KT616693.1|Spug_DNA        TTATTTGATCTGTATTTATTACAACTATTCTATTATTATTATCTTTGCCTGTATTAGCTG  540

OP001985|Ppen_DNA          GTGCTATTACTATATTGTTAACTGATCGAAATTTTAATACTTCTTTTTTTGATCCAGCTG  600
HQ924602.1|Pala_DNA        GAGCTATTACTATATTATTAACTGATCGTAATTTTAATACTTCTTTTTTTGATCCAGCTG  600
KT616693.1|Spug_DNA        GAGCTATTACTATATTGTTAACTGATCGCAATTTTAATACTTCTTTTTTTGATCCTGCTG  600
```
