# Supplementary material for: Transcriptome analysis of the spider Phonotimpus pennimani reveals novel toxin transcripts
Source: J Venom Anim Toxins Incl Trop Dis. 2023 Jan 23;29:e20220031. doi: 10.1590/1678-9199-JVATITD-2022-0031 (PMC9881743; doi:10.1590/1678-9199-JVATITD-2022-0031)
Supplement: Additional file 2. [file 1678-9199-jvatitd-29-e20220031-s2.pdf]

J Venom Anim Toxins incl Trop Dis
https://doi.org/10.1590/1678-9199-JVATITD-2022-0031

Supplementary Material to "Transcriptome analysis of the spider *Phonotimpus pennimani* reveals novel toxin transcripts"

Additional file 2. Trinotate annotation of 212 *Phonotimpus pennimani* transcripts and their correspondence to Blastx and Blastp data after keyword filtering.

X.gene_id	transcript_id	sprot_Top_BLASTX_hit	sprot_Top_BLASTP_hit
TRINITY_DN21619_c0_g1	TRINITY_DN21619_c0_g1_i1	ABCG1_HUMAN^ABCG1_HUMAN^Q:5-394,H:541-673^48.12%ID^E:1.5e-34^RecName: Full=ATP-binding cassette sub-family G member 1	ABCG1_HUMAN^ABCG1_HUMAN^Q:2-131,H:541-673^48.12%ID^E:6.9e-35^RecName: Full=ATP-binding cassette sub-family G member 1
TRINITY_DN56926_c0_g1	TRINITY_DN56926_c0_g1_i1	CRVP1_DISTY^CRVP1_DISTY^Q:7-195,H:40-100^44.444%ID^E:7.55e-11^RecName: Full=Cysteine-rich venom protein DIS1	.
TRINITY_DN70170_c0_g1	TRINITY_DN70170_c0_g1_i1	A411_LOXHI^A411_LOXHI^Q:7-387,H:147-277^45.802%ID^E:1.16e-32^RecName: Full=Phospholipase D LhSicTox-alphaIV1i	A411_LOXHI^A411_LOXHI^Q:3-129,H:147-277^45.802%ID^E:6.79e-33^RecName: Full=Phospholipase D LhSicTox-alphaIV1i
TRINITY_DN5976_c0_g2	TRINITY_DN5976_c0_g2_i1	SMC4_HUMAN^SMC4_HUMAN^Q:4-510,H:90-259^58.824%ID^E:2.21e-63^RecName: Full=Structural maintenance of chromosomes protein 4	SMC4_HUMAN^SMC4_HUMAN^Q:2-170,H:90-259^58.824%ID^E:1.99e-63^RecName: Full=Structural maintenance of chromosomes protein 4
TRINITY_DN11384_c0_g1	TRINITY_DN11384_c0_g1_i1	NRP1_MOUSE^NRP1_MOUSE^Q:1303-1809,H:431-605^27.624%ID^E:9.41e-07^RecName: Full=Neuropilin-1	NRP1_MOUSE^NRP1_MOUSE^Q:435-603,H:431-605^27.624%ID^E:3.93e-07^RecName: Full=Neuropilin-1
TRINITY_DN85506_c0_g1	TRINITY_DN85506_c0_g1_i1	TCPH_HUMAN^TCPH_HUMAN^Q:54-1634,H:2-528^78.368%ID^E:0^RecName: Full=T-complex protein 1 subunit eta	TCPH_MOUSE^TCPH_MOUSE^Q:3-541,H:2-540^77.365%ID^E:0^RecName: Full=T-complex protein 1 subunit eta
TRINITY_DN3043_c0_g1	TRINITY_DN3043_c0_g1_i1	TENA_DROME^TENA_DROME^Q:6-1784,H:2327-2966^39.531%ID^E:2.09e-140^RecName: Full=Teneurin-a	TENA_DROME^TENA_DROME^Q:2-594,H:2327-2966^39.531%ID^E:1.75e-148^RecName: Full=Teneurin-a
TRINITY_DN13842_c0_g1	TRINITY_DN13842_c0_g1_i1	.	.
TRINITY_DN2658_c0_g1	TRINITY_DN2658_c0_g1_i1	.	VP164_LYCMC^VP164_LYCMC^Q:8-97,H:4-90^35.87%ID^E:1.33e-06^RecName: Full=Venom protein 164
TRINITY_DN2753_c0_g1	TRINITY_DN2753_c0_g1_i1	CP1A5_CHICK^CP1A5_CHICK^Q:186-1562,H:49-520^27.629%ID^E:9.23e-55^RecName: Full=Cytochrome P450 1A5	CP1A5_CHICK^CP1A5_CHICK^Q:38-496,H:49-520^27.629%ID^E:2.97e-55^RecName: Full=Cytochrome P450 1A5
TRINITY_DN25619_c0_g1	TRINITY_DN25619_c0_g1_i1	FA5_PSETE^FA5_PSETE^Q:13-120,H:1423-1458^50%ID^E:2.73e-06^RecName: Full=Coagulation factor V	FA5_PSETE^FA5_PSETE^Q:5-40,H:1423-1458^50%ID^E:2.46e-06^RecName: Full=Coagulation factor V
TRINITY_DN43845_c0_g1	TRINITY_DN43845_c0_g1_i1	MEP1B_HUMAN^MEP1B_HUMAN^Q:18-95,H:149-174^65.385%ID^E:6.31e-06^RecName: Full=Meprin A subunit beta	.
TRINITY_DN20626_c0_g1	TRINITY_DN20626_c0_g1_i1	RAB28_MOUSE^RAB28_MOUSE^Q:3-656,H:10-221^52.294%ID^E:3.35e-75^RecName: Full=Ras-related protein Rab-28	RAB28_MOUSE^RAB28_MOUSE^Q:1-190,H:10-199^57.368%ID^E:8.31e-76^RecName: Full=Ras-related protein Rab-28
TRINITY_DN57579_c0_g1	TRINITY_DN57579_c0_g1_i1	MEP1B_HUMAN^MEP1B_HUMAN^Q:9-101,H:148-178^58.065%ID^E:7.15e-07^RecName: Full=Meprin A subunit beta	.
TRINITY_DN92504_c0_g1	TRINITY_DN92504_c0_g1_i1	MDR3_HUMAN^MDR3_HUMAN^Q:1-177,H:797-855^45.763%ID^E:5.22e-14^RecName: Full=Phosphatidylcholine translocator ABCB4	.
TRINITY_DN74254_c0_g1	TRINITY_DN74254_c0_g1_i1	SCFD1_MOUSE^SCFD1_MOUSE^Q:15-335,H:2-110^70.642%ID^E:9.91e-49^RecName: Full=Sec1 family domain-containing protein 1	SCFD1_MOUSE^SCFD1_MOUSE^Q:5-111,H:2-110^70.642%ID^E:8.92e-49^RecName: Full=Sec1 family domain-containing protein 1
TRINITY_DN72512_c0_g1	TRINITY_DN72512_c0_g1_i1	LRP6_HUMAN^LRP6_HUMAN^Q:1-456,H:1015-1167^40.523%ID^E:1.25e-34^RecName: Full=Low-density lipoprotein receptor-related protein 6	LRP6_HUMAN^LRP6_HUMAN^Q:1-152,H:1015-1167^40.523%ID^E:1.12e-34^RecName: Full=Low-density lipoprotein receptor-related protein 6
TRINITY_DN6962_c0_g1	TRINITY_DN6962_c0_g1_i1	LAT2_MOUSE^LAT2_MOUSE^Q:170-1621,H:15-500^54.303%ID^E:7.91e-148^RecName: Full=Large neutral amino acids transporter small subunit 2	LAT2_MOUSE^LAT2_MOUSE^Q:7-490,H:15-500^54.303%ID^E:9.37e-164^RecName: Full=Large neutral amino acids transporter small subunit 2
TRINITY_DN6984_c0_g1	TRINITY_DN6984_c0_g1_i1	LRP6_HUMAN^LRP6_HUMAN^Q:4-111,H:1305-1338^55.556%ID^E:9.8e-07^RecName: Full=Low-density lipoprotein receptor-related protein 6	.
TRINITY_DN4853_c0_g1	TRINITY_DN4853_c0_g1_i1	SMC1A_HUMAN^SMC1A_HUMAN^Q:3-392,H:1096-1225^77.692%ID^E:2.54e-69^RecName: Full=Structural maintenance of chromosomes protein 1A	SMC1A_HUMAN^SMC1A_HUMAN^Q:1-130,H:1096-1225^77.692%ID^E:3.24e-70^RecName: Full=Structural maintenance of chromosomes protein 1A
TRINITY_DN36672_c0_g1	TRINITY_DN36672_c0_g1_i1	CERS1_HUMAN^CERS1_HUMAN^Q:263-580,H:70-174^49.057%ID^E:4.87e-26^RecName: Full=Ceramide synthase 1	CERS1_HUMAN^CERS1_HUMAN^Q:88-193,H:70-174^49.057%ID^E:4.39e-26^RecName: Full=Ceramide synthase 1
TRINITY_DN99797_c0_g1	TRINITY_DN99797_c0_g1_i1	LRP6_MOUSE^LRP6_MOUSE^Q:17-148,H:588-630^56.818%ID^E:7.59e-10^RecName: Full=Low-density lipoprotein receptor-related protein 6	.
TRINITY_DN22755_c0_g1	TRINITY_DN22755_c0_g1_i1	ABCG1_HUMAN^ABCG1_HUMAN^Q:1-213,H:204-274^60.563%ID^E:1.69e-24^RecName: Full=ATP-binding cassette sub-family G member 1	.
TRINITY_DN3661_c0_g2	TRINITY_DN3661_c0_g2_i1	TENM_DROME^TENM_DROME^Q:28-585,H:2512-2702^38.22%ID^E:3.66e-35^RecName: Full=Teneurin-m	TENM_DROME^TENM_DROME^Q:10-195,H:2512-2702^38.22%ID^E:5.47e-37^RecName: Full=Teneurin-m
TRINITY_DN14684_c0_g1	TRINITY_DN14684_c0_g1_i1	VM3M1_NAJMO^VM3M1_NAJMO^Q:4-420,H:395-533^49.645%ID^E:4.65e-37^RecName: Full=Snake venom metalloproteinase-disintegrin-like mocarhagin	VM3M1_NAJMO^VM3M1_NAJMO^Q:2-140,H:395-533^49.645%ID^E:4.19e-37^RecName: Full=Snake venom metalloproteinase-disintegrin-like mocarhagin

X.gene_id	transcript_id	sprot_Top_BLASTX_hit	sprot_Top_BLASTP_hit
TRINITY_DN13195_c1_g2	TRINITY_DN13195_c1_g2_i1	CTHA2_CONVC^CTHA2_CONVC^Q:253-552,H:45-144^52%ID^E:3.36e-32^RecName: Full=Thyrostimulin alpha-2 subunit	CTHA2_CONVC^CTHA2_CONVC^Q:26-125,H:45-144^52%ID^E:8.02e-34^RecName: Full=Thyrostimulin alpha-2 subunit
TRINITY_DN71263_c0_g1	TRINITY_DN71263_c0_g1_i1	VMPA_LOXIN^VMPA_LOXIN^Q:111-746,H:35-248^47.248%ID^E:1.8e-50^RecName: Full=Astacin-like metalloprotease toxin 1	VMPA_LOXIN^VMPA_LOXIN^Q:37-248,H:35-248^47.248%ID^E:9.13e-51^RecName: Full=Astacin-like metalloprotease toxin 1
TRINITY_DN87515_c0_g1	TRINITY_DN87515_c0_g1_i1	TRIP4_HUMAN^TRIP4_HUMAN^Q:86-1288,H:131-575^42.731%ID^E:5.16e-106^RecName: Full=Activating signal cointegrator 1	TRIP4_HUMAN^TRIP4_HUMAN^Q:21-398,H:163-575^44.076%ID^E:1.63e-106^RecName: Full=Activating signal cointegrator 1
TRINITY_DN63119_c0_g1	TRINITY_DN63119_c0_g1_i1	DYN1_RAT^DYN1_RAT^Q:3-134,H:52-95^79.545%ID^E:8.93e-19^RecName: Full=Dynamin-1	.
TRINITY_DN63137_c0_g1	TRINITY_DN63137_c0_g1_i1	RAC1_RAT^RAC1_RAT^Q:3-242,H:69-148^67.5%ID^E:1.12e-36^RecName: Full=Ras-related C3 botulinum toxin substrate 1	.
TRINITY_DN4292_c0_g1	TRINITY_DN4292_c0_g1_i1	LPHN_DROME^LPHN_DROME^Q:491-1843,H:270-777^31.579%ID^E:1.04e-64^RecName: Full=Latrophilin Cirl	LPHN_DROME^LPHN_DROME^Q:85-537,H:270-779^32.022%ID^E:2.4e-73^RecName: Full=Latrophilin Cirl
TRINITY_DN4292_c0_g1	TRINITY_DN4292_c0_g1_i2	LPHN_DROME^LPHN_DROME^Q:744-2096,H:270-777^31.579%ID^E:3.52e-64^RecName: Full=Latrophilin Cirl	LPHN_DROME^LPHN_DROME^Q:161-613,H:270-779^32.022%ID^E:3.35e-72^RecName: Full=Latrophilin Cirl
TRINITY_DN4292_c0_g1	TRINITY_DN4292_c0_g1_i3	LPHN_DROME^LPHN_DROME^Q:681-2039,H:270-779^31.461%ID^E:3.14e-64^RecName: Full=Latrophilin Cirl	LPHN_DROME^LPHN_DROME^Q:140-592,H:270-779^32.022%ID^E:3.55e-72^RecName: Full=Latrophilin Cirl
TRINITY_DN1415_c2_g1	TRINITY_DN1415_c2_g1_i1	TX610_LYCSI^TX610_LYCSI^Q:106-282,H:17-75^45.763%ID^E:1.15e-11^RecName: Full=U6-lycotoxin-Ls1b	.
TRINITY_DN3501_c0_g2	TRINITY_DN3501_c0_g2_i1	DYN1_BOVIN^DYN1_BOVIN^Q:2-160,H:688-740^62.264%ID^E:2.08e-08^RecName: Full=Dynamin-1	.
TRINITY_DN2901_c0_g1	TRINITY_DN2901_c0_g1_i1	VP164_LYCMC^VP164_LYCMC^Q:86-313,H:4-77^35.526%ID^E:3.25e-07^RecName: Full=Venom protein 164	VP164_LYCMC^VP164_LYCMC^Q:8-83,H:4-77^35.526%ID^E:8.64e-08^RecName: Full=Venom protein 164
TRINITY_DN6883_c0_g1	TRINITY_DN6883_c0_g1_i1	TXAG8_AGEOR^TXAG8_AGEOR^Q:91-372,H:1-89^31.915%ID^E:4.92e-08^RecName: Full=U8-agatoxin-Ao1a	.
TRINITY_DN72274_c0_g1	TRINITY_DN72274_c0_g1_i1	DJC17_BOVIN^DJC17_BOVIN^Q:66-938,H:6-298^50.662%ID^E:8.11e-86^RecName: Full=DnaJ homolog subfamily C member 17	DJC17_BOVIN^DJC17_BOVIN^Q:3-293,H:6-298^50.662%ID^E:1.89e-86^RecName: Full=DnaJ homolog subfamily C member 17
TRINITY_DN74998_c0_g1	TRINITY_DN74998_c0_g1_i1	SIR1_RAT^SIR1_RAT^Q:3-329,H:55-163^78.899%ID^E:1.63e-56^RecName: Full=NAD-dependent protein deacetylase sirtuin-1	SIR1_RAT^SIR1_RAT^Q:1-109,H:55-163^78.899%ID^E:1.47e-56^RecName: Full=NAD-dependent protein deacetylase sirtuin-1
TRINITY_DN9325_c0_g1	TRINITY_DN9325_c0_g1_i1	RAB7L_PONAB^RAB7L_PONAB^Q:1-207,H:62-130^60.87%ID^E:1.36e-27^RecName: Full=Ras-related protein Rab-7L1	.
TRINITY_DN53591_c0_g1	TRINITY_DN53591_c0_g1_i1	RAC2_HUMAN^RAC2_HUMAN^Q:9-152,H:145-192^68.75%ID^E:3.11e-16^RecName: Full=Ras-related C3 botulinum toxin substrate 2	.
TRINITY_DN78624_c0_g1	TRINITY_DN78624_c0_g1_i1	B1R2_LOXSN^B1R2_LOXSN^Q:2-169,H:34-88^39.286%ID^E:2.07e-09^RecName: Full=Phospholipase D LspiSicTox-betaIE1ii	.
TRINITY_DN90502_c0_g1	TRINITY_DN90502_c0_g1_i1	GGA3_MOUSE^GGA3_MOUSE^Q:85-348,H:168-255^52.273%ID^E:7.98e-22^RecName: Full=ADP-ribosylation factor-binding protein GGA3	GGA1_MOUSE^GGA1_MOUSE^Q:1-102,H:143-241^46.078%ID^E:4.2e-18^RecName: Full=ADP-ribosylation factor-binding protein GGA1
TRINITY_DN9179_c0_g1	TRINITY_DN9179_c0_g1_i1	VMPA_LOXIN^VMPA_LOXIN^Q:21-692,H:26-248^42.795%ID^E:4.07e-50^RecName: Full=Astacin-like metalloprotease toxin 1	VMPA_LOXIN^VMPA_LOXIN^Q:7-230,H:26-248^42.795%ID^E:2.09e-50^RecName: Full=Astacin-like metalloprotease toxin 1
TRINITY_DN10861_c0_g2	TRINITY_DN10861_c0_g2_i1	ABCA3_HUMAN^ABCA3_HUMAN^Q:1-531,H:1528-1701^47.458%ID^E:1.9e-51^RecName: Full=ATP-binding cassette sub-family A member 3	ABCA3_HUMAN^ABCA3_HUMAN^Q:1-177,H:1528-1701^47.458%ID^E:7.51e-52^RecName: Full=ATP-binding cassette sub-family A member 3
TRINITY_DN13481_c0_g2	TRINITY_DN13481_c0_g2_i1	LAT2_RABIT^LAT2_RABIT^Q:3-812,H:187-461^52.727%ID^E:8.92e-84^RecName: Full=Large neutral amino acids transporter small subunit 2	LAT2_RABIT^LAT2_RABIT^Q:1-270,H:187-461^52.727%ID^E:3.6e-88^RecName: Full=Large neutral amino acids transporter small subunit 2
TRINITY_DN5330_c0_g1	TRINITY_DN5330_c0_g1_i1	VMPA_LOXIN^VMPA_LOXIN^Q:129-764,H:35-248^45.872%ID^E:4.54e-52^RecName: Full=Astacin-like metalloprotease toxin 1	VMPA_LOXIN^VMPA_LOXIN^Q:43-254,H:35-248^45.872%ID^E:1.18e-52^RecName: Full=Astacin-like metalloprotease toxin 1
TRINITY_DN13355_c0_g2	TRINITY_DN13355_c0_g2_i1	LAT2_RABIT^LAT2_RABIT^Q:16-375,H:23-142^58.333%ID^E:2.44e-24^RecName: Full=Large neutral amino acids transporter small subunit 2	LAT2_RABIT^LAT2_RABIT^Q:6-125,H:23-142^58.333%ID^E:1.14e-39^RecName: Full=Large neutral amino acids transporter small subunit 2
TRINITY_DN10743_c0_g1	TRINITY_DN10743_c0_g1_i1	FMO2_RAT^FMO2_RAT^Q:10-369,H:380-499^43.333%ID^E:1.08e-25^RecName: Full=Dimethylaniline monooxygenase [N-oxide-forming] 2	FMO2_RAT^FMO2_RAT^Q:4-123,H:380-499^43.333%ID^E:6.3e-26^RecName: Full=Dimethylaniline monooxygenase [N-oxide-forming] 2
TRINITY_DN85826_c0_g1	TRINITY_DN85826_c0_g1_i1	VMPA_LOXIN^VMPA_LOXIN^Q:38-745,H:15-248^45.267%ID^E:1.13e-57^RecName: Full=Astacin-like metalloprotease toxin 1	VMPA_LOXIN^VMPA_LOXIN^Q:13-248,H:15-248^45.267%ID^E:3.86e-58^RecName: Full=Astacin-like metalloprotease toxin 1
TRINITY_DN6170_c0_g2	TRINITY_DN6170_c0_g2_i1	VM3K_NAJKA^VM3K_NAJKA^Q:8-310,H:104-196^40.196%ID^E:4.53e-13^RecName: Full=Hemorrhagic metalloproteinase-disintegrin-like kaouthiagin	VM3K_NAJKA^VM3K_NAJKA^Q:3-103,H:104-196^40.196%ID^E:4.08e-13^RecName: Full=Hemorrhagic metalloproteinase-disintegrin-like kaouthiagin
TRINITY_DN6136_c0_g1	TRINITY_DN6136_c0_g1_i1	TCPB_BOVIN^TCPB_BOVIN^Q:71-1672,H:4-535^64.981%ID^E:0^RecName: Full=T-complex protein 1 subunit beta	TCPB_BOVIN^TCPB_BOVIN^Q:24-557,H:4-535^64.981%ID^E:0^RecName: Full=T-complex protein 1 subunit beta
TRINITY_DN16191_c0_g1	TRINITY_DN16191_c0_g1_i1	LRP6_MOUSE^LRP6_MOUSE^Q:7-180,H:219-276^48.276%ID^E:7.11e-13^RecName: Full=Low-density lipoprotein receptor-related protein 6	.
TRINITY_DN20786_c0_g1	TRINITY_DN20786_c0_g1_i1	PA2B2_DABRR^PA2B2_DABRR^Q:256-645,H:4-127^35.115%ID^E:3.51e-17^RecName: Full=Basic phospholipase A2 Drk-b2	PA2B2_DABRR^PA2B2_DABRR^Q:9-138,H:4-127^35.115%ID^E:2.73e-18^RecName: Full=Basic phospholipase A2 Drk-b2
TRINITY_DN63365_c0_g1	TRINITY_DN63365_c0_g1_i1	TCPH_HUMAN^TCPH_HUMAN^Q:74-3,H:429-452^83.333%ID^E:3.72e-07^RecName: Full=T-complex protein 1 subunit eta	.
TRINITY_DN3265_c0_g1	TRINITY_DN3265_c0_g1_i1	RAC1_RAT^RAC1_RAT^Q:153-728,H:1-192^91.146%ID^E:1.71e-128^RecName: Full=Ras-related C3 botulinum toxin substrate 1	RAC1_RAT^RAC1_RAT^Q:1-192,H:1-192^91.146%ID^E:4.26e-131^RecName: Full=Ras-related C3 botulinum toxin substrate 1
TRINITY_DN3265_c0_g1	TRINITY_DN3265_c0_g1_i1	RAC1_RAT^RAC1_RAT^Q:153-728,H:1-192^91.146%ID^E:1.71e-128^RecName: Full=Ras-related C3 botulinum toxin substrate 1	.
TRINITY_DN57890_c0_g1	TRINITY_DN57890_c0_g1_i1	LRP6_HUMAN^LRP6_HUMAN^Q:3-170,H:472-527^44.643%ID^E:2.49e-12^RecName: Full=Low-density lipoprotein receptor-related protein 6	.

X.gene_id	transcript_id	sprot_Top_BLASTX_hit	sprot_Top_BLASTP_hit
TRINITY_DN6072_c0_g1	TRINITY_DN6072_c0_g1_i1	ABCGN_DICDI^ABCGN_DICDI^Q:6-365,H:126-245^51.667%ID^E:2.76e-33^RecName: Full=ABC transporter G family member 23	ABCGN_DICDI^ABCGN_DICDI^Q:2-121,H:126-245^51.667%ID^E:2.48e-33^RecName: Full=ABC transporter G family member 23
TRINITY_DN199_c0_g2	TRINITY_DN199_c0_g2_i1	CO3_LETCA^CO3_LETCA^Q:52-4884,H:96-1646^28.332%ID^E:1.57e-171^RecName: Full=Complement C3	CO3_LETCA^CO3_LETCA^Q:18-1628,H:96-1646^28.435%ID^E:1.53e-174^RecName: Full=Complement C3
TRINITY_DN123_c0_g1	TRINITY_DN123_c0_g1_i1	TXZ01_LYCSI^TXZ01_LYCSI^Q:131-508,H:1-114^45.238%ID^E:6.85e-27^RecName: Full=Toxin-like structure LSTX-D1	TXZ01_LYCSI^TXZ01_LYCSI^Q:4-124,H:1-109^46.281%ID^E:9.93e-28^RecName: Full=Toxin-like structure LSTX-D1
TRINITY_DN123_c0_g1	TRINITY_DN123_c0_g1_i2	TX152_LYCSI^TX152_LYCSI^Q:122-499,H:1-110^36.508%ID^E:1.32e-13^RecName: Full=U1-lycotoxin-Ls1jj	TX501_LYCSI^TX501_LYCSI^Q:4-126,H:1-114^34.146%ID^E:1.33e-16^RecName: Full=U5-lycotoxin-Ls1a
TRINITY_DN112_c0_g1	TRINITY_DN112_c0_g1_i1	TX606_LYCSI^TX606_LYCSI^Q:129-290,H:24-75^42.593%ID^E:1.29e-07^RecName: Full=U6-lycotoxin-Ls1f	.
TRINITY_DN112_c0_g1	TRINITY_DN112_c0_g1_i3	TX711_LYCSI^TX711_LYCSI^Q:129-284,H:25-74^42.308%ID^E:8.4e-07^RecName: Full=U7-lycotoxin-Ls1c	.
TRINITY_DN170_c0_g1	TRINITY_DN170_c0_g1_i4	TX602_LYCSI^TX602_LYCSI^Q:79-246,H:19-73^42.857%ID^E:1.41e-08^RecName: Full=U6-lycotoxin-Ls1c	.
TRINITY_DN170_c0_g1	TRINITY_DN170_c0_g1_i5	TX602_LYCSI^TX602_LYCSI^Q:102-269,H:19-73^42.857%ID^E:2.72e-08^RecName: Full=U6-lycotoxin-Ls1c	.
TRINITY_DN170_c0_g1	TRINITY_DN170_c0_g1_i6	TX602_LYCSI^TX602_LYCSI^Q:79-252,H:19-75^41.379%ID^E:2.56e-08^RecName: Full=U6-lycotoxin-Ls1c	.
TRINITY_DN170_c0_g1	TRINITY_DN170_c0_g1_i3	TX606_LYCSI^TX606_LYCSI^Q:79-246,H:19-73^42.857%ID^E:1.31e-08^RecName: Full=U6-lycotoxin-Ls1f	TX606_LYCSI^TX606_LYCSI^Q:10-82,H:1-73^39.726%ID^E:5.76e-13^RecName: Full=U6-lycotoxin-Ls1f
TRINITY_DN170_c0_g1	TRINITY_DN170_c0_g1_i8	TX602_LYCSI^TX602_LYCSI^Q:79-252,H:19-75^44.828%ID^E:3.4e-10^RecName: Full=U6-lycotoxin-Ls1c	.
TRINITY_DN170_c0_g1	TRINITY_DN170_c0_g1_i9	TX830_LYCSI^TX830_LYCSI^Q:28-234,H:1-71^45.07%ID^E:3.91e-07^RecName: Full=U9-lycotoxin-Ls1a	.
TRINITY_DN170_c0_g1	TRINITY_DN170_c0_g1_i12	TX711_LYCSI^TX711_LYCSI^Q:79-249,H:20-75^43.86%ID^E:1.44e-08^RecName: Full=U7-lycotoxin-Ls1c	.
TRINITY_DN132_c0_g1	TRINITY_DN132_c0_g1_i1	VMPA_LOXIN^VMPA_LOXIN^Q:177-803,H:35-248^42.056%ID^E:2.27e-49^RecName: Full=Astacin-like metalloprotease toxin 1	VMPA_LOXIN^VMPA_LOXIN^Q:6-245,H:7-248^38.776%ID^E:9.19e-51^RecName: Full=Astacin-like metalloprotease toxin 1
TRINITY_DN132_c0_g1	TRINITY_DN132_c0_g1_i1	VMPA_LOXIN^VMPA_LOXIN^Q:177-803,H:35-248^42.056%ID^E:2.27e-49^RecName: Full=Astacin-like metalloprotease toxin 1	.
TRINITY_DN71494_c0_g1	TRINITY_DN71494_c0_g1_i1	.	LRP6_HUMAN^LRP6_HUMAN^Q:10-68,H:1460-1538^40.506%ID^E:6.61e-06^RecName: Full=Low-density lipoprotein receptor-related protein 6
TRINITY_DN37401_c0_g1	TRINITY_DN37401_c0_g1_i1	VMPA2_LOXIN^VMPA2_LOXIN^Q:4-210,H:113-181^53.623%ID^E:1.76e-23^RecName: Full=Astacin-like metalloprotease toxin 2	.
TRINITY_DN88384_c0_g1	TRINITY_DN88384_c0_g1_i1	ABCG1_HUMAN^ABCG1_HUMAN^Q:13-108,H:312-343^56.25%ID^E:2.23e-06^RecName: Full=ATP-binding cassette sub-family G member 1	.
TRINITY_DN28857_c0_g1	TRINITY_DN28857_c0_g1_i1	CTHA2_CONVC^CTHA2_CONVC^Q:28-165,H:47-92^67.391%ID^E:3.83e-17^RecName: Full=Thyrostimulin alpha-2 subunit	.
TRINITY_DN1757_c0_g1	TRINITY_DN1757_c0_g1_i1	CSTN1_DROME^CSTN1_DROME^Q:135-2777,H:9-935^38.285%ID^E:0^RecName: Full=Calsyntenin-1	CSTN1_DROME^CSTN1_DROME^Q:3-883,H:9-935^38.285%ID^E:0^RecName: Full=Calsyntenin-1
TRINITY_DN44322_c0_g1	TRINITY_DN44322_c0_g1_i1	SMD_IXOSC^SMD_IXOSC^Q:139-303,H:3-59^52.632%ID^E:3.28e-11^RecName: Full=Sphingomyelin phosphodiesterase D	.
TRINITY_DN53341_c0_g1	TRINITY_DN53341_c0_g1_i1	SIR1_RAT^SIR1_RAT^Q:2-844,H:158-467^44.201%ID^E:7.64e-65^RecName: Full=NAD-dependent protein deacetylase sirtuin-1	SIR1_RAT^SIR1_RAT^Q:1-279,H:158-465^44.654%ID^E:3.78e-70^RecName: Full=NAD-dependent protein deacetylase sirtuin-1
TRINITY_DN54585_c0_g1	TRINITY_DN54585_c0_g1_i1	AMD_RAT^AMD_RAT^Q:2-253,H:249-330^51.19%ID^E:3.86e-22^RecName: Full=Peptidyl-glycine alpha-amidating monooxygenase	AMD_RAT^AMD_RAT^Q:1-84,H:249-330^51.19%ID^E:3.48e-22^RecName: Full=Peptidyl-glycine alpha-amidating monooxygenase
TRINITY_DN1195_c0_g1	TRINITY_DN1195_c0_g1_i1	TXK04_LYCSI^TXK04_LYCSI^Q:71-325,H:18-102^43.678%ID^E:4.67e-19^RecName: Full=U20-lycotoxin-Ls1d	.
TRINITY_DN1195_c0_g1	TRINITY_DN1195_c0_g1_i2	TXAG7_AGEOR^TXAG7_AGEOR^Q:183-338,H:45-96^40.385%ID^E:1.45e-09^RecName: Full=U7-agatoxin-Ao1a	.
TRINITY_DN17893_c0_g1	TRINITY_DN17893_c0_g1_i1	VKT52_ANESU^VKT52_ANESU^Q:3-98,H:23-54^59.375%ID^E:1.14e-08^RecName: Full=KappaPI-actitoxin-Avd3a	.
TRINITY_DN17897_c0_g1	TRINITY_DN17897_c0_g1_i1	VKT52_ANESU^VKT52_ANESU^Q:1-99,H:25-57^60.606%ID^E:2.23e-09^RecName: Full=KappaPI-actitoxin-Avd3a	.
TRINITY_DN2383_c0_g1	TRINITY_DN2383_c0_g1_i4	AFLK_ASPPU^AFLK_ASPPU^Q:2-127,H:511-552^35.714%ID^E:7.93e-06^RecName: Full=Versicolorin B synthase	.
TRINITY_DN515_c0_g1	TRINITY_DN515_c0_g1_i1	TCPD_RAT^TCPD_RAT^Q:87-1682,H:5-537^72.285%ID^E:0^RecName: Full=T-complex protein 1 subunit delta	TCPD_RAT^TCPD_RAT^Q:7-531,H:12-537^73.055%ID^E:0^RecName: Full=T-complex protein 1 subunit delta
TRINITY_DN542_c0_g1	TRINITY_DN542_c0_g1_i1	VMPA_LOXIN^VMPA_LOXIN^Q:170-766,H:50-248^46.766%ID^E:3.2e-52^RecName: Full=Astacin-like metalloprotease toxin 1	VMPA_LOXIN^VMPA_LOXIN^Q:3-244,H:4-248^42.915%ID^E:5.04e-55^RecName: Full=Astacin-like metalloprotease toxin 1
TRINITY_DN542_c0_g1	TRINITY_DN542_c0_g1_i1	VMPA_LOXIN^VMPA_LOXIN^Q:170-766,H:50-248^46.766%ID^E:3.2e-52^RecName: Full=Astacin-like metalloprotease toxin 1	.
TRINITY_DN501_c0_g1	TRINITY_DN501_c0_g1_i1	KCY_XENLA^KCY_XENLA^Q:134-694,H:2-192^56.771%ID^E:4.37e-75^RecName: Full=UMP-CMP kinase	KCY_XENLA^KCY_XENLA^Q:15-201,H:2-192^56.771%ID^E:1.97e-76^RecName: Full=UMP-CMP kinase
TRINITY_DN547_c0_g1	TRINITY_DN547_c0_g1_i4	TXE04_LYCSI^TXE04_LYCSI^Q:218-400,H:22-83^39.683%ID^E:6.15e-06^RecName: Full=U14-lycotoxin-Ls1b	TXE04_LYCSI^TXE04_LYCSI^Q:73-133,H:22-83^39.683%ID^E:3.78e-06^RecName: Full=U14-lycotoxin-Ls1b

X.gene_id	transcript_id	sprot_Top_BLASTX_hit	sprot_Top_BLASTP_hit
TRINITY_DN547_c0_g1	TRINITY_DN547_c0_g1_i1	TXE04_LYCSI^TXE04_LYCSI^Q:131-313,H:22-83^39.683%ID^E:8.65e-06^RecName: Full=U14-lycotoxin-Ls1b	.
TRINITY_DN547_c0_g1	TRINITY_DN547_c0_g1_i2	TXAG7_AGEOR^TXAG7_AGEOR^Q:71-319,H:1-84^27.059%ID^E:5.53e-06^RecName: Full=U7-agatoxin-Ao1a	TXAG7_AGEOR^TXAG7_AGEOR^Q:24-106,H:1-84^27.059%ID^E:2.62e-06^RecName: Full=U7-agatoxin-Ao1a
TRINITY_DN547_c0_g3	TRINITY_DN547_c0_g3_i1	TXAG7_AGEOR^TXAG7_AGEOR^Q:71-346,H:1-90^28.421%ID^E:5.79e-07^RecName: Full=U7-agatoxin-Ao1a	TXAG7_AGEOR^TXAG7_AGEOR^Q:24-115,H:1-90^28.421%ID^E:1.23e-07^RecName: Full=U7-agatoxin-Ao1a
TRINITY_DN5823_c0_g1	TRINITY_DN5823_c0_g1_i4	TXAG8_AGEOR^TXAG8_AGEOR^Q:122-424,H:1-89^36.275%ID^E:5.61e-08^RecName: Full=U8-agatoxin-Ao1a	.
TRINITY_DN5823_c0_g1	TRINITY_DN5823_c0_g1_i4	TXAG8_AGEOR^TXAG8_AGEOR^Q:122-424,H:1-89^36.275%ID^E:5.61e-08^RecName: Full=U8-agatoxin-Ao1a	TXAG8_AGEOR^TXAG8_AGEOR^Q:1-101,H:1-89^36.275%ID^E:9.96e-09^RecName: Full=U8-agatoxin-Ao1a
TRINITY_DN5823_c0_g1	TRINITY_DN5823_c0_g1_i1	TXAG8_AGEOR^TXAG8_AGEOR^Q:122-409,H:1-89^35.417%ID^E:2.27e-07^RecName: Full=U8-agatoxin-Ao1a	.
TRINITY_DN5823_c0_g1	TRINITY_DN5823_c0_g1_i2	TXAG8_AGEOR^TXAG8_AGEOR^Q:140-427,H:1-89^35.417%ID^E:2.55e-07^RecName: Full=U8-agatoxin-Ao1a	.
TRINITY_DN5823_c0_g1	TRINITY_DN5823_c0_g1_i3	TXAG8_AGEOR^TXAG8_AGEOR^Q:305-430,H:50-89^52.381%ID^E:2.47e-06^RecName: Full=U8-agatoxin-Ao1a	TXAG8_AGEOR^TXAG8_AGEOR^Q:62-103,H:50-89^52.381%ID^E:2.74e-07^RecName: Full=U8-agatoxin-Ao1a
TRINITY_DN2037_c0_g1	TRINITY_DN2037_c0_g1_i2	PDE2_CROAD^PDE2_CROAD^Q:338-2464,H:81-803^36.022%ID^E:4.7e-144^RecName: Full=Venom phosphodiesterase 2	PDE2_CROAD^PDE2_CROAD^Q:82-790,H:81-803^36.022%ID^E:1.73e-144^RecName: Full=Venom phosphodiesterase 2
TRINITY_DN2037_c0_g2	TRINITY_DN2037_c0_g2_i1	PDE2_CROAD^PDE2_CROAD^Q:2-1804,H:192-803^35.182%ID^E:9.19e-116^RecName: Full=Venom phosphodiesterase 2	PDE2_CROAD^PDE2_CROAD^Q:1-601,H:192-803^35.182%ID^E:6.2e-116^RecName: Full=Venom phosphodiesterase 2
TRINITY_DN69_c0_g5	TRINITY_DN69_c0_g5_i1	VMPA_LOXIN^VMPA_LOXIN^Q:154-774,H:35-248^40.465%ID^E:1.72e-46^RecName: Full=Astacin-like metalloprotease toxin 1	VMPA_LOXIN^VMPA_LOXIN^Q:32-238,H:35-248^40.465%ID^E:1.32e-47^RecName: Full=Astacin-like metalloprotease toxin 1
TRINITY_DN69_c0_g5	TRINITY_DN69_c0_g5_i2	VMPA_LOXIN^VMPA_LOXIN^Q:8-592,H:50-248^41.709%ID^E:6.37e-48^RecName: Full=Astacin-like metalloprotease toxin 1	VMPA_LOXIN^VMPA_LOXIN^Q:3-197,H:50-248^41.709%ID^E:1.96e-48^RecName: Full=Astacin-like metalloprotease toxin 1
TRINITY_DN44_c0_g1	TRINITY_DN44_c0_g1_i4	VKT5_ANEVI^VKT5_ANEVI^Q:759-920,H:16-71^62.5%ID^E:3.69e-15^RecName: Full=U-actitoxin-Avd3h	VKT5_ANEVI^VKT5_ANEVI^Q:253-317,H:16-82^55.224%ID^E:3.28e-16^RecName: Full=U-actitoxin-Avd3h
TRINITY_DN44_c0_g1	TRINITY_DN44_c0_g1_i5	VKT5_ANEVI^VKT5_ANEVI^Q:717-911,H:16-82^55.224%ID^E:9.21e-16^RecName: Full=U-actitoxin-Avd3h	PPN1_CAEEL^PPN1_CAEEL^Q:94-294,H:1382-1556^31.527%ID^E:7.09e-19^RecName: Full=Papilin
TRINITY_DN44_c0_g1	TRINITY_DN44_c0_g1_i6	VKT5_ANEVI^VKT5_ANEVI^Q:717-911,H:16-82^55.224%ID^E:9.43e-16^RecName: Full=U-actitoxin-Avd3h	PPN1_CAEEL^PPN1_CAEEL^Q:94-294,H:1382-1556^31.527%ID^E:7.09e-19^RecName: Full=Papilin
TRINITY_DN44_c0_g1	TRINITY_DN44_c0_g1_i1	VKT3_VIPAA^VKT3_VIPAA^Q:243-434,H:24-90^50.746%ID^E:1.69e-14^RecName: Full=Kunitz-type serine protease inhibitor 3	VKT1_ANTEL^VKT1_ANTEL^Q:87-140,H:5-58^55.556%ID^E:6.99e-16^RecName: Full=KappaPI-actitoxin-Ael3a
TRINITY_DN44_c0_g1	TRINITY_DN44_c0_g1_i2	VKT5_ANEVI^VKT5_ANEVI^Q:759-953,H:16-82^55.224%ID^E:8.94e-16^RecName: Full=U-actitoxin-Avd3h	VKT5_ANEVI^VKT5_ANEVI^Q:253-317,H:16-82^55.224%ID^E:3.67e-16^RecName: Full=U-actitoxin-Avd3h
TRINITY_DN44_c0_g1	TRINITY_DN44_c0_g1_i3	VKT5_ANEVI^VKT5_ANEVI^Q:759-953,H:16-82^55.224%ID^E:8.04e-16^RecName: Full=U-actitoxin-Avd3h	VKT5_ANEVI^VKT5_ANEVI^Q:253-317,H:16-82^55.224%ID^E:3.03e-16^RecName: Full=U-actitoxin-Avd3h
TRINITY_DN44_c0_g1	TRINITY_DN44_c0_g1_i8	VKT5_ANEVI^VKT5_ANEVI^Q:759-953,H:16-82^55.224%ID^E:8.76e-16^RecName: Full=U-actitoxin-Avd3h	VKT5_ANEVI^VKT5_ANEVI^Q:253-317,H:16-82^55.224%ID^E:3.67e-16^RecName: Full=U-actitoxin-Avd3h
TRINITY_DN81251_c0_g1	TRINITY_DN81251_c0_g1_i1	PDE1_CROAD^PDE1_CROAD^Q:3-125,H:282-322^48.78%ID^E:5.07e-06^RecName: Full=Venom phosphodiesterase 1	.
TRINITY_DN39630_c0_g1	TRINITY_DN39630_c0_g1_i1	VKTA_ANEVI^VKTA_ANEVI^Q:2-154,H:21-71^47.059%ID^E:1.45e-12^RecName: Full=U-actitoxin-Avd3m	.
TRINITY_DN86083_c0_g1	TRINITY_DN86083_c0_g1_i1	LAT2_RABIT^LAT2_RABIT^Q:1-240,H:240-319^71.25%ID^E:7.01e-43^RecName: Full=Large neutral amino acids transporter small subunit 2	LAT2_RABIT^LAT2_RABIT^Q:1-80,H:240-319^71.25%ID^E:9.59e-34^RecName: Full=Large neutral amino acids transporter small subunit 2
TRINITY_DN13049_c0_g2	TRINITY_DN13049_c0_g2_i1	VPS11_MOUSE^VPS11_MOUSE^Q:3-308,H:472-574^69.903%ID^E:1.6e-41^RecName: Full=Vacuolar protein sorting-associated protein 11 homolog	VPS11_MOUSE^VPS11_MOUSE^Q:1-102,H:472-574^69.903%ID^E:1.44e-41^RecName: Full=Vacuolar protein sorting-associated protein 11 homolog
TRINITY_DN52369_c0_g1	TRINITY_DN52369_c0_g1_i1	DYN1_HUMAN^DYN1_HUMAN^Q:318-1751,H:8-493^39.095%ID^E:6.13e-103^RecName: Full=Dynamin-1	DYN1_BOVIN^DYN1_BOVIN^Q:53-530,H:8-493^39.3%ID^E:6.38e-106^RecName: Full=Dynamin-1
TRINITY_DN4491_c0_g2	TRINITY_DN4491_c0_g2_i1	VPS11_MOUSE^VPS11_MOUSE^Q:5-214,H:105-175^50%ID^E:3.28e-17^RecName: Full=Vacuolar protein sorting-associated protein 11 homolog	.
TRINITY_DN20837_c0_g1	TRINITY_DN20837_c0_g1_i1	SCFD1_RAT^SCFD1_RAT^Q:17-1432,H:166-635^64.286%ID^E:0^RecName: Full=Sec1 family domain-containing protein 1	SCFD1_RAT^SCFD1_RAT^Q:6-477,H:166-635^64.286%ID^E:0^RecName: Full=Sec1 family domain-containing protein 1
TRINITY_DN20911_c0_g1	TRINITY_DN20911_c0_g1_i1	TXAG8_AGEOR^TXAG8_AGEOR^Q:69-344,H:1-89^31.522%ID^E:7.13e-10^RecName: Full=U8-agatoxin-Ao1a	.
TRINITY_DN88624_c0_g1	TRINITY_DN88624_c0_g1_i1	MDR3_HUMAN^MDR3_HUMAN^Q:41-427,H:348-477^53.846%ID^E:3.67e-38^RecName: Full=Phosphatidylcholine translocator ABCB4	.
TRINITY_DN53244_c0_g1	TRINITY_DN53244_c0_g1_i1	ARI1A_HUMAN^ARI1A_HUMAN^Q:612-872,H:1634-1720^51.724%ID^E:7.63e-26^RecName: Full=AT-rich interactive domain-containing protein 1A	ARI1A_HUMAN^ARI1A_HUMAN^Q:204-290,H:1634-1720^51.724%ID^E:6.87e-26^RecName: Full=AT-rich interactive domain-containing protein 1A
TRINITY_DN12705_c0_g1	TRINITY_DN12705_c0_g1_i1	VMPA_LOXIN^VMPA_LOXIN^Q:133-780,H:35-248^36.036%ID^E:4.48e-32^RecName: Full=Astacin-like metalloprotease toxin 1	LCE_ORYLA^LCE_ORYLA^Q:22-246,H:51-271^35.47%ID^E:5.56e-35^RecName: Full=Low choriolytic enzyme
TRINITY_DN86326_c0_g1	TRINITY_DN86326_c0_g1_i1	VPP1_MOUSE^VPP1_MOUSE^Q:5-805,H:569-839^56.044%ID^E:1.37e-84^RecName: Full=V-type proton ATPase 116 kDa subunit a isoform 1	VPP1_MOUSE^VPP1_MOUSE^Q:2-268,H:569-839^56.777%ID^E:6.37e-93^RecName: Full=V-type proton ATPase 116 kDa subunit a isoform 1
TRINITY_DN37744_c0_g1	TRINITY_DN37744_c0_g1_i1	CERS1_MOUSE^CERS1_MOUSE^Q:1-372,H:225-348^37.903%ID^E:4.58e-21^RecName: Full=Ceramide synthase 1	CERS1_MOUSE^CERS1_MOUSE^Q:1-122,H:225-346^38.525%ID^E:2.21e-21^RecName: Full=Ceramide synthase 1
TRINITY_DN69965_c0_g1	TRINITY_DN69965_c0_g1_i1	ABCC9_MOUSE^ABCC9_MOUSE^Q:3-329,H:689-812^54.839%ID^E:1.01e-37^RecName: Full=ATP-binding cassette sub-family C member 9	ABCC9_MOUSE^ABCC9_MOUSE^Q:1-109,H:689-812^54.839%ID^E:9.05e-38^RecName: Full=ATP-binding cassette sub-family C member 9

X.gene_id	transcript_id	sprot_Top_BLASTX_hit	sprot_Top_BLASTP_hit
TRINITY_DN221_c0_g1	TRINITY_DN221_c0_g1_i1	TXCA_CAEEX^TXCA_CAEEX^Q:49-315,H:6-87^37.079%ID^E:2.03e-11^RecName: Full=U3-aranetoxin-Ce1a	TXCA_CAEEX^TXCA_CAEEX^Q:5-93,H:6-87^37.079%ID^E:2.99e-12^RecName: Full=U3-aranetoxin-Ce1a
TRINITY_DN69052_c0_g1	TRINITY_DN69052_c0_g1_i1	NATT4_THANI^NATT4_THANI^Q:228-650,H:77-206^31.25%ID^E:9.45e-06^RecName: Full=Natterin-4	NATT4_THANI^NATT4_THANI^Q:14-154,H:77-206^31.25%ID^E:2.7e-06^RecName: Full=Natterin-4
TRINITY_DN24565_c0_g1	TRINITY_DN24565_c0_g1_i1	VPS11_MOUSE^VPS11_MOUSE^Q:3-434,H:766-908^60.417%ID^E:9.77e-59^RecName: Full=Vacuolar protein sorting-associated protein 11 homolog	VPS11_MOUSE^VPS11_MOUSE^Q:1-144,H:766-908^60.417%ID^E:8.79e-59^RecName: Full=Vacuolar protein sorting-associated protein 11 homolog
TRINITY_DN801_c0_g1	TRINITY_DN801_c0_g1_i1	VMPA_LOXIN^VMPA_LOXIN^Q:137-766,H:35-248^44.444%ID^E:2.89e-49^RecName: Full=Astacin-like metalloprotease toxin 1	VMPA_LOXIN^VMPA_LOXIN^Q:32-241,H:35-248^44.444%ID^E:5.51e-50^RecName: Full=Astacin-like metalloprotease toxin 1
TRINITY_DN863_c0_g1	TRINITY_DN863_c0_g1_i2	.	SVMI1_CERCE^SVMI1_CERCE^Q:3-110,H:94-201^20.37%ID^E:1.43e-06^RecName: Full=Snake venom metalloprotease inhibitor 02A10
TRINITY_DN14281_c0_g2	TRINITY_DN14281_c0_g2_i1	TENM_DROME^TENM_DROME^Q:31-741,H:2461-2702^38.43%ID^E:1.32e-42^RecName: Full=Teneurin-m	TENM_DROME^TENM_DROME^Q:11-247,H:2461-2702^38.43%ID^E:2.65e-49^RecName: Full=Teneurin-m
TRINITY_DN14201_c0_g1	TRINITY_DN14201_c0_g1_i1	AGRL1_BOVIN^AGRL1_BOVIN^Q:3-323,H:1010-1111^33.636%ID^E:7.69e-07^RecName: Full=Adhesion G protein-coupled receptor L1	AGRL1_BOVIN^AGRL1_BOVIN^Q:1-107,H:1010-1111^33.636%ID^E:6.92e-07^RecName: Full=Adhesion G protein-coupled receptor L1
TRINITY_DN3344_c0_g1	TRINITY_DN3344_c0_g1_i1	AGRL3_HUMAN^AGRL3_HUMAN^Q:700-2502,H:498-1109^32.227%ID^E:1.32e-74^RecName: Full=Adhesion G protein-coupled receptor L3	AGRL1_MOUSE^AGRL1_MOUSE^Q:158-771,H:450-1097^32.934%ID^E:5.7e-93^RecName: Full=Adhesion G protein-coupled receptor L1
TRINITY_DN1375_c0_g1	TRINITY_DN1375_c0_g1_i3	CP1A2_MOUSE^CP1A2_MOUSE^Q:72-173,H:37-70^50%ID^E:3.64e-06^RecName: Full=Cytochrome P450 1A2	.
TRINITY_DN370_c0_g1	TRINITY_DN370_c0_g1_i1	VKT1_TRILK^VKT1_TRILK^Q:1330-1863,H:28-165^35.955%ID^E:8.03e-26^RecName: Full=Kunitz-type U19-barytoxin-Tl1a	VKT1_TRILK^VKT1_TRILK^Q:444-621,H:28-165^35.955%ID^E:6.82e-26^RecName: Full=Kunitz-type U19-barytoxin-Tl1a
TRINITY_DN370_c0_g1	TRINITY_DN370_c0_g1_i1	VKT1_TRILK^VKT1_TRILK^Q:1330-1863,H:28-165^35.955%ID^E:8.03e-26^RecName: Full=Kunitz-type U19-barytoxin-Tl1a	.
TRINITY_DN370_c0_g1	TRINITY_DN370_c0_g1_i1	VKT1_TRILK^VKT1_TRILK^Q:1330-1863,H:28-165^35.955%ID^E:8.03e-26^RecName: Full=Kunitz-type U19-barytoxin-Tl1a	.
TRINITY_DN370_c0_g1	TRINITY_DN370_c0_g1_i1	VKT1_TRILK^VKT1_TRILK^Q:1330-1863,H:28-165^35.955%ID^E:8.03e-26^RecName: Full=Kunitz-type U19-barytoxin-Tl1a	.
TRINITY_DN370_c0_g1	TRINITY_DN370_c0_g1_i9	VKT1_ANTAF^VKT1_ANTAF^Q:1876-2046,H:1-57^63.158%ID^E:1.25e-16^RecName: Full=PI-actitoxin-Axm2a	VKT1_ANTAF^VKT1_ANTAF^Q:626-682,H:1-57^63.158%ID^E:1.08e-16^RecName: Full=PI-actitoxin-Axm2a
TRINITY_DN370_c0_g1	TRINITY_DN370_c0_g1_i9	VKT1_ANTAF^VKT1_ANTAF^Q:1876-2046,H:1-57^63.158%ID^E:1.25e-16^RecName: Full=PI-actitoxin-Axm2a	.
TRINITY_DN370_c0_g1	TRINITY_DN370_c0_g1_i9	VKT1_ANTAF^VKT1_ANTAF^Q:1876-2046,H:1-57^63.158%ID^E:1.25e-16^RecName: Full=PI-actitoxin-Axm2a	.
TRINITY_DN370_c0_g1	TRINITY_DN370_c0_g1_i9	VKT1_ANTAF^VKT1_ANTAF^Q:1876-2046,H:1-57^63.158%ID^E:1.25e-16^RecName: Full=PI-actitoxin-Axm2a	.
TRINITY_DN370_c0_g1	TRINITY_DN370_c0_g1_i9	VKT1_ANTAF^VKT1_ANTAF^Q:1876-2046,H:1-57^63.158%ID^E:1.25e-16^RecName: Full=PI-actitoxin-Axm2a	.
TRINITY_DN55120_c0_g1	TRINITY_DN55120_c0_g1_i1	BNIP3_MOUSE^BNIP3_MOUSE^Q:101-604,H:6-180^37.433%ID^E:2.73e-17^RecName: Full=BCL2/adenovirus E1B 19 kDa protein-interacting protein 3	BNIP3_HUMAN^BNIP3_HUMAN^Q:20-201,H:61-252^35.122%ID^E:2.06e-19^RecName: Full=BCL2/adenovirus E1B 19 kDa protein-interacting protein 3
TRINITY_DN1905_c0_g1	TRINITY_DN1905_c0_g1_i1	VKT1_TRILK^VKT1_TRILK^Q:165-575,H:24-166^42.657%ID^E:6.11e-27^RecName: Full=Kunitz-type U19-barytoxin-Tl1a	VKT1_TRILK^VKT1_TRILK^Q:55-191,H:24-166^42.657%ID^E:2.47e-27^RecName: Full=Kunitz-type U19-barytoxin-Tl1a
TRINITY_DN44696_c0_g1	TRINITY_DN44696_c0_g1_i1	LRP6_HUMAN^LRP6_HUMAN^Q:12-137,H:651-692^73.81%ID^E:2.61e-15^RecName: Full=Low-density lipoprotein receptor-related protein 6	.
TRINITY_DN13632_c0_g1	TRINITY_DN13632_c0_g1_i1	TENM_DROME^TENM_DROME^Q:35-685,H:2463-2672^37.327%ID^E:1.08e-32^RecName: Full=Teneurin-m	TENM_DROME^TENM_DROME^Q:12-228,H:2463-2672^37.327%ID^E:4.2e-38^RecName: Full=Teneurin-m
TRINITY_DN4128_c0_g1	TRINITY_DN4128_c0_g1_i2	PA2_SCOVI^PA2_SCOVI^Q:264-515,H:57-142^37.931%ID^E:6.98e-11^RecName: Full=Phospholipase A2 Scol/Pla	PA2_URTCR^PA2_URTCR^Q:57-99,H:42-84^41.86%ID^E:4.06e-07^RecName: Full=Phospholipase A2 A2-actitoxin-Ucs2a
TRINITY_DN7026_c0_g1	TRINITY_DN7026_c0_g1_i1	AGRL2_HUMAN^AGRL2_HUMAN^Q:225-1238,H:691-1016^23.276%ID^E:1.76e-16^RecName: Full=Adhesion G protein-coupled receptor L2	AGRL2_HUMAN^AGRL2_HUMAN^Q:75-412,H:691-1016^23.276%ID^E:1.59e-16^RecName: Full=Adhesion G protein-coupled receptor L2
TRINITY_DN11425_c0_g1	TRINITY_DN11425_c0_g1_i1	TENM_DROME^TENM_DROME^Q:6-1313,H:2261-2700^35.333%ID^E:7.36e-94^RecName: Full=Teneurin-m	TENM_DROME^TENM_DROME^Q:2-446,H:2261-2709^35.512%ID^E:1.06e-97^RecName: Full=Teneurin-m
TRINITY_DN52201_c0_g1	TRINITY_DN52201_c0_g1_i1	SLB_CROHD^SLB_CROHD^Q:96-341,H:9-90^40.476%ID^E:1.4e-09^RecName: Full=Snaclec CHH-B subunit beta	SLB_CROHD^SLB_CROHD^Q:32-113,H:9-90^40.476%ID^E:1.26e-09^RecName: Full=Snaclec CHH-B subunit beta
TRINITY_DN20540_c0_g1	TRINITY_DN20540_c0_g1_i1	VPS11_MOUSE^VPS11_MOUSE^Q:3-233,H:244-323^48.75%ID^E:5.45e-20^RecName: Full=Vacuolar protein sorting-associated protein 11 homolog	.
TRINITY_DN1660_c0_g1	TRINITY_DN1660_c0_g1_i1	TXAG4_AGEOR^TXAG4_AGEOR^Q:112-243,H:21-67^57.447%ID^E:1.46e-11^RecName: Full=U4-agatoxin-Ao1a	.
TRINITY_DN435_c0_g1	TRINITY_DN435_c0_g1_i1	SNF4_ARATH^SNF4_ARATH^Q:1568-1831,H:12-101^35.165%ID^E:1.41e-09^RecName: Full=Sucrose nonfermenting 4-like protein	SVMI1_CERCE^SVMI1_CERCE^Q:232-415,H:46-220^20.541%ID^E:1.64e-08^RecName: Full=Snake venom metalloprotease inhibitor 02A10
TRINITY_DN435_c0_g1	TRINITY_DN435_c0_g1_i2	SNF4_ARATH^SNF4_ARATH^Q:1841-2104,H:12-101^35.165%ID^E:1.66e-09^RecName: Full=Sucrose nonfermenting 4-like protein	SVMI1_CERCE^SVMI1_CERCE^Q:273-456,H:46-220^20.541%ID^E:1.35e-08^RecName: Full=Snake venom metalloprotease inhibitor 02A10
TRINITY_DN421_c0_g1	TRINITY_DN421_c0_g1_i1	ABCG1_MOUSE^ABCG1_MOUSE^Q:256-2049,H:72-666^50.82%ID^E:0^RecName: Full=ATP-binding cassette sub-family G member 1	ABCG1_HUMAN^ABCG1_HUMAN^Q:86-683,H:72-678^50.971%ID^E:0^RecName: Full=ATP-binding cassette sub-family G member 1
TRINITY_DN37199_c0_g1	TRINITY_DN37199_c0_g1_i1	VMPA_LOXIN^VMPA_LOXIN^Q:135-782,H:35-254^41.071%ID^E:1.73e-43^RecName: Full=Astacin-like metalloprotease toxin 1	VMPA_LOXIN^VMPA_LOXIN^Q:45-260,H:35-254^41.071%ID^E:2.87e-44^RecName: Full=Astacin-like metalloprotease toxin 1
TRINITY_DN8949_c0_g1	TRINITY_DN8949_c0_g1_i1	DNA2_ACAPL^DNA2_ACAPL^Q:5-232,H:92-173^48.78%ID^E:1.6e-18^RecName: Full=Plancitoxin-1	.

X.gene_id	transcript_id	sprot_Top_BLASTX_hit	sprot_Top_BLASTP_hit
TRINITY_DN62771_c0_g1	TRINITY_DN62771_c0_g1_i1	VPS11_MOUSE^VPS11_MOUSE^Q:1-120,H:374-413^72.5%ID^E:2.51e-13^RecName: Full=Vacuolar protein sorting-associated protein 11 homolog	.
TRINITY_DN86582_c0_g1	TRINITY_DN86582_c0_g1_i1	LRP6_MOUSE^LRP6_MOUSE^Q:10-579,H:815-998^57.068%ID^E:8.08e-70^RecName: Full=Low-density lipoprotein receptor-related protein 6	LRP6_MOUSE^LRP6_MOUSE^Q:4-193,H:815-998^57.068%ID^E:7.27e-70^RecName: Full=Low-density lipoprotein receptor-related protein 6
TRINITY_DN14752_c0_g1	TRINITY_DN14752_c0_g1_i1	LAT2_RABIT^LAT2_RABIT^Q:3-377,H:253-377^58.4%ID^E:2.23e-39^RecName: Full=Large neutral amino acids transporter small subunit 2	LAT2_RABIT^LAT2_RABIT^Q:1-125,H:253-377^58.4%ID^E:2.01e-39^RecName: Full=Large neutral amino acids transporter small subunit 2
TRINITY_DN23154_c0_g1	TRINITY_DN23154_c0_g1_i1	LRP6_HUMAN^LRP6_HUMAN^Q:7-330,H:1180-1288^46.789%ID^E:4.91e-29^RecName: Full=Low-density lipoprotein receptor-related protein 6	LRP6_HUMAN^LRP6_HUMAN^Q:3-110,H:1180-1288^46.789%ID^E:4.42e-29^RecName: Full=Low-density lipoprotein receptor-related protein 6
TRINITY_DN49616_c0_g1	TRINITY_DN49616_c0_g1_i1	VPS11_MOUSE^VPS11_MOUSE^Q:5-166,H:637-690^44.444%ID^E:2.23e-08^RecName: Full=Vacuolar protein sorting-associated protein 11 homolog	.
TRINITY_DN37538_c0_g1	TRINITY_DN37538_c0_g1_i1	ABCG1_HUMAN^ABCG1_HUMAN^Q:12-701,H:446-675^44.828%ID^E:1.14e-63^RecName: Full=ATP-binding cassette sub-family G member 1	ABCG1_HUMAN^ABCG1_HUMAN^Q:4-233,H:446-675^44.828%ID^E:1.02e-63^RecName: Full=ATP-binding cassette sub-family G member 1
TRINITY_DN37026_c0_g1	TRINITY_DN37026_c0_g1_i1	RAC1_RAT^RAC1_RAT^Q:46-522,H:4-159^44.848%ID^E:1.54e-37^RecName: Full=Ras-related C3 botulinum toxin substrate 1	RAC1_RAT^RAC1_RAT^Q:16-174,H:4-159^44.848%ID^E:1.39e-37^RecName: Full=Ras-related C3 botulinum toxin substrate 1
TRINITY_DN52251_c0_g1	TRINITY_DN52251_c0_g1_i1	VPP1_HUMAN^VPP1_HUMAN^Q:70-876,H:169-437^65.428%ID^E:3.65e-131^RecName: Full=V-type proton ATPase 116 kDa subunit a isoform 1	VPP1_HUMAN^VPP1_HUMAN^Q:24-292,H:169-437^65.428%ID^E:3.29e-131^RecName: Full=V-type proton ATPase 116 kDa subunit a isoform 1
TRINITY_DN86886_c0_g1	TRINITY_DN86886_c0_g1_i1	VM3BE_BOTER^VM3BE_BOTER^Q:2-754,H:290-541^44.488%ID^E:1.12e-56^RecName: Full=Zinc metalloproteinase-disintegrin-like berythractivase	VM3BE_BOTER^VM3BE_BOTER^Q:1-251,H:290-541^44.488%ID^E:3.6e-61^RecName: Full=Zinc metalloproteinase-disintegrin-like berythractivase
TRINITY_DN23299_c0_g1	TRINITY_DN23299_c0_g1_i1	CRVP_TELDH^CRVP_TELDH^Q:326-826,H:28-190^42.857%ID^E:6.6e-36^RecName: Full=Cysteine-rich venom protein TEL1	CRVP_TELDH^CRVP_TELDH^Q:48-214,H:28-190^42.857%ID^E:9.42e-37^RecName: Full=Cysteine-rich venom protein TEL1
TRINITY_DN6284_c0_g1	TRINITY_DN6284_c0_g1_i1	VKT1_TRILK^VKT1_TRILK^Q:63-455,H:12-156^38.356%ID^E:4.81e-27^RecName: Full=Kunitz-type U19-barytoxin-Tl1a	VKT1_TRILK^VKT1_TRILK^Q:21-151,H:12-156^38.356%ID^E:2.55e-27^RecName: Full=Kunitz-type U19-barytoxin-Tl1a
TRINITY_DN6284_c0_g1	TRINITY_DN6284_c0_g1_i1	VKT1_TRILK^VKT1_TRILK^Q:63-455,H:12-156^38.356%ID^E:4.81e-27^RecName: Full=Kunitz-type U19-barytoxin-Tl1a	.
TRINITY_DN6284_c0_g1	TRINITY_DN6284_c0_g1_i2	VKT1_TRILK^VKT1_TRILK^Q:63-455,H:12-156^38.356%ID^E:3.65e-27^RecName: Full=Kunitz-type U19-barytoxin-Tl1a	VKT1_TRILK^VKT1_TRILK^Q:21-151,H:12-156^38.356%ID^E:1.3e-27^RecName: Full=Kunitz-type U19-barytoxin-Tl1a
TRINITY_DN6284_c0_g1	TRINITY_DN6284_c0_g1_i2	VKT1_TRILK^VKT1_TRILK^Q:63-455,H:12-156^38.356%ID^E:3.65e-27^RecName: Full=Kunitz-type U19-barytoxin-Tl1a	.
TRINITY_DN1800_c0_g1	TRINITY_DN1800_c0_g1_i1	COPZ1_BOVIN^COPZ1_BOVIN^Q:87-608,H:1-174^60.345%ID^E:8.13e-70^RecName: Full=Coatomer subunit zeta-1	COPZ1_BOVIN^COPZ1_BOVIN^Q:1-177,H:1-177^60.452%ID^E:1.28e-82^RecName: Full=Coatomer subunit zeta-1
TRINITY_DN17685_c0_g1	TRINITY_DN17685_c0_g1_i1	ABCG1_HUMAN^ABCG1_HUMAN^Q:2-232,H:448-524^58.442%ID^E:3.86e-28^RecName: Full=ATP-binding cassette sub-family G member 1	.
TRINITY_DN2497_c0_g1	TRINITY_DN2497_c0_g1_i1	B1Q_LOXIN^B1Q_LOXIN^Q:201-1094,H:1-304^50.489%ID^E:3.01e-102^RecName: Full=Phospholipase D LiSicTox-betaID1	B1Q_LOXIN^B1Q_LOXIN^Q:13-311,H:1-305^50.325%ID^E:2.79e-103^RecName: Full=Phospholipase D LiSicTox-betaID1
TRINITY_DN2497_c0_g2	TRINITY_DN2497_c0_g2_i1	B1R2_LOXSN^B1R2_LOXSN^Q:173-3,H:158-214^57.895%ID^E:2.85e-17^RecName: Full=Phospholipase D LspiSicTox-betaIE1ii	.
TRINITY_DN36352_c0_g1	TRINITY_DN36352_c0_g1_i1	PN16_PHONI^PN16_PHONI^Q:126-470,H:4-115^36.522%ID^E:7.14e-22^RecName: Full=U24-ctenitoxin-Pn1a	PN16_PHONI^PN16_PHONI^Q:42-156,H:4-115^36.522%ID^E:2.45e-22^RecName: Full=U24-ctenitoxin-Pn1a
TRINITY_DN85561_c0_g1	TRINITY_DN85561_c0_g1_i1	PN16_PHONI^PN16_PHONI^Q:174-494,H:4-108^42.056%ID^E:9.37e-22^RecName: Full=U24-ctenitoxin-Pn1a	PN16_PHONI^PN16_PHONI^Q:20-126,H:4-108^42.056%ID^E:3.88e-23^RecName: Full=U24-ctenitoxin-Pn1a
TRINITY_DN85561_c0_g1	TRINITY_DN85561_c0_g1_i1	PN16_PHONI^PN16_PHONI^Q:174-494,H:4-108^42.056%ID^E:9.37e-22^RecName: Full=U24-ctenitoxin-Pn1a	.
TRINITY_DN37840_c0_g1	TRINITY_DN37840_c0_g1_i1	IGFBP_CUPSA^IGFBP_CUPSA^Q:85-735,H:20-242^50.222%ID^E:6.44e-68^RecName: Full=Insulin-like growth factor-binding protein-related protein 1	IGFBP_CUPSA^IGFBP_CUPSA^Q:29-248,H:20-245^50.439%ID^E:5.19e-70^RecName: Full=Insulin-like growth factor-binding protein-related protein 1
TRINITY_DN45431_c0_g1	TRINITY_DN45431_c0_g1_i1	PN16_PHONI^PN16_PHONI^Q:99-1,H:10-42^54.545%ID^E:7.71e-06^RecName: Full=U24-ctenitoxin-Pn1a	.
TRINITY_DN71800_c0_g1	TRINITY_DN71800_c0_g1_i1	TLLP_PHONI^TLLP_PHONI^Q:6-173,H:140-196^65.517%ID^E:2e-17^RecName: Full=Techylectin-like protein	.
TRINITY_DN20772_c0_g1	TRINITY_DN20772_c0_g1_i1	IGFBP_CUPSA^IGFBP_CUPSA^Q:85-873,H:1-261^81.749%ID^E:3.68e-161^RecName: Full=Insulin-like growth factor-binding protein-related protein 1	IGFBP_CUPSA^IGFBP_CUPSA^Q:1-263,H:1-261^81.749%ID^E:2.33e-161^RecName: Full=Insulin-like growth factor-binding protein-related protein 1
TRINITY_DN1723_c0_g1	TRINITY_DN1723_c0_g1_i1	SIBD1_CUPSA^SIBD1_CUPSA^Q:10-291,H:11-97^42.553%ID^E:1.01e-16^RecName: Full=Single insulin-like growth factor-binding domain protein-1	.
TRINITY_DN1723_c0_g1	TRINITY_DN1723_c0_g1_i2	SIBD1_CUPSA^SIBD1_CUPSA^Q:48-344,H:1-97^46.465%ID^E:2.7e-22^RecName: Full=Single insulin-like growth factor-binding domain protein-1	SIBD1_CUPSA^SIBD1_CUPSA^Q:1-99,H:1-97^46.465%ID^E:5.02e-23^RecName: Full=Single insulin-like growth factor-binding domain protein-1
TRINITY_DN17_c0_g1	TRINITY_DN17_c0_g1_i1	PN16_PHONI^PN16_PHONI^Q:108-503,H:1-127^45.588%ID^E:4.84e-24^RecName: Full=U24-ctenitoxin-Pn1a	PN16_PHONI^PN16_PHONI^Q:20-151,H:1-127^45.588%ID^E:5.44e-25^RecName: Full=U24-ctenitoxin-Pn1a
TRINITY_DN17_c0_g1	TRINITY_DN17_c0_g1_i2	PN16_PHONI^PN16_PHONI^Q:117-515,H:1-127^43.478%ID^E:2.27e-22^RecName: Full=U24-ctenitoxin-Pn1a	PN16_PHONI^PN16_PHONI^Q:20-152,H:1-127^43.478%ID^E:1.55e-23^RecName: Full=U24-ctenitoxin-Pn1a
TRINITY_DN17_c0_g2	TRINITY_DN17_c0_g2_i1	PN16_PHONI^PN16_PHONI^Q:94-486,H:1-128^69.466%ID^E:5.84e-59^RecName: Full=U24-ctenitoxin-Pn1a	PN16_PHONI^PN16_PHONI^Q:32-162,H:1-128^69.466%ID^E:8.04e-60^RecName: Full=U24-ctenitoxin-Pn1a
TRINITY_DN71_c0_g1	TRINITY_DN71_c0_g1_i2	TX31_PHONI^TX31_PHONI^Q:55-306,H:1-83^41.667%ID^E:2.82e-07^RecName: Full=Kappa-ctenitoxin-Pn1a	.
TRINITY_DN71_c0_g1	TRINITY_DN71_c0_g1_i3	TX3A_PHONI^TX3A_PHONI^Q:55-294,H:1-78^49.383%ID^E:4.6e-16^RecName: Full=U6-ctenitoxin-Pn1a	.
TRINITY_DN52826_c0_g1	TRINITY_DN52826_c0_g1_i1	SIBD2_CUPSA^SIBD2_CUPSA^Q:261-401,H:28-74^57.447%ID^E:1.15e-10^RecName: Full=Single insulin-like growth factor-binding domain protein-2	SIBD2_CUPSA^SIBD2_CUPSA^Q:39-115,H:24-97^41.558%ID^E:1.53e-11^RecName: Full=Single insulin-like growth factor-binding domain protein-2

X.gene_id	transcript_id	sprot_Top_BLASTX_hit	sprot_Top_BLASTP_hit
TRINITY_DN16439_c0_g1	TRINITY_DN16439_c0_g1_i1	PN16_PHONI^PN16_PHONI^Q:89-484,H:2-128^46.617%ID^E:8.4e-28^RecName: Full=U24-ctenitoxin-Pn1a	PN16_PHONI^PN16_PHONI^Q:29-161,H:1-128^47.015%ID^E:1.07e-28^RecName: Full=U24-ctenitoxin-Pn1a
TRINITY_DN270_c0_g1	TRINITY_DN270_c0_g1_i1	PN16_PHONI^PN16_PHONI^Q:87-455,H:2-127^32.031%ID^E:3.01e-12^RecName: Full=U24-ctenitoxin-Pn1a	PN16_PHONI^PN16_PHONI^Q:22-131,H:2-113^32.456%ID^E:9.95e-13^RecName: Full=U24-ctenitoxin-Pn1a
TRINITY_DN270_c0_g1	TRINITY_DN270_c0_g1_i1	PN16_PHONI^PN16_PHONI^Q:87-455,H:2-127^32.031%ID^E:3.01e-12^RecName: Full=U24-ctenitoxin-Pn1a	.
TRINITY_DN1379_c0_g1	TRINITY_DN1379_c0_g1_i1	TXC20_CUPSA^TXC20_CUPSA^Q:78-263,H:3-65^52.381%ID^E:1.23e-14^RecName: Full=Toxin CSTX-20	TXC20_CUPSA^TXC20_CUPSA^Q:26-104,H:3-84^52.439%ID^E:1.9e-19^RecName: Full=Toxin CSTX-20
TRINITY_DN1379_c0_g1	TRINITY_DN1379_c0_g1_i1	TXC20_CUPSA^TXC20_CUPSA^Q:78-263,H:3-65^52.381%ID^E:1.23e-14^RecName: Full=Toxin CSTX-20	.
TRINITY_DN1379_c0_g1	TRINITY_DN1379_c0_g1_i2	TXC20_CUPSA^TXC20_CUPSA^Q:78-263,H:3-65^52.381%ID^E:2.34e-14^RecName: Full=Toxin CSTX-20	.
TRINITY_DN1379_c0_g1	TRINITY_DN1379_c0_g1_i2	TXC20_CUPSA^TXC20_CUPSA^Q:78-263,H:3-65^52.381%ID^E:2.34e-14^RecName: Full=Toxin CSTX-20	TXC20_CUPSA^TXC20_CUPSA^Q:26-104,H:3-84^52.439%ID^E:1.9e-19^RecName: Full=Toxin CSTX-20
TRINITY_DN1913_c0_g1	TRINITY_DN1913_c0_g1_i1	PN16_PHONI^PN16_PHONI^Q:194-559,H:1-122^41.803%ID^E:5.24e-24^RecName: Full=U24-ctenitoxin-Pn1a	PN16_PHONI^PN16_PHONI^Q:18-139,H:1-122^41.803%ID^E:4.78e-25^RecName: Full=U24-ctenitoxin-Pn1a
TRINITY_DN1645_c0_g1	TRINITY_DN1645_c0_g1_i1	TXC20_CUPSA^TXC20_CUPSA^Q:249-491,H:1-82^30.488%ID^E:7.62e-07^RecName: Full=Toxin CSTX-20	TXC20_CUPSA^TXC20_CUPSA^Q:20-100,H:1-82^30.488%ID^E:1.11e-07^RecName: Full=Toxin CSTX-20
TRINITY_DN1645_c0_g1	TRINITY_DN1645_c0_g1_i2	TXC20_CUPSA^TXC20_CUPSA^Q:276-518,H:1-82^30.488%ID^E:1.06e-06^RecName: Full=Toxin CSTX-20	TXC20_CUPSA^TXC20_CUPSA^Q:29-109,H:1-82^30.488%ID^E:1.89e-07^RecName: Full=Toxin CSTX-20
TRINITY_DN55842_c0_g1	TRINITY_DN55842_c0_g1_i1	PN47_PHONI^PN47_PHONI^Q:144-872,H:1-244^46.939%ID^E:4.95e-71^RecName: Full=U21-ctenitoxin-Pn1a	PN47_PHONI^PN47_PHONI^Q:44-286,H:1-244^46.939%ID^E:2.14e-71^RecName: Full=U21-ctenitoxin-Pn1a
TRINITY_DN37840_c0_g1	TRINITY_DN37840_c0_g1_i1	IGFBP_CUPSA^IGFBP_CUPSA^Q:85-735,H:20-242^50.222%ID^E:6.44e-68^RecName: Full=Insulin-like growth factor-binding protein-related protein 1	IGFBP_CUPSA^IGFBP_CUPSA^Q:29-248,H:20-245^50.439%ID^E:5.19e-70^RecName: Full=Insulin-like growth factor-binding protein-related protein 1
TRINITY_DN20772_c0_g1	TRINITY_DN20772_c0_g1_i1	IGFBP_CUPSA^IGFBP_CUPSA^Q:85-873,H:1-261^81.749%ID^E:3.68e-161^RecName: Full=Insulin-like growth factor-binding protein-related protein 1	IGFBP_CUPSA^IGFBP_CUPSA^Q:1-263,H:1-261^81.749%ID^E:2.33e-161^RecName: Full=Insulin-like growth factor-binding protein-related protein 1
TRINITY_DN1723_c0_g1	TRINITY_DN1723_c0_g1_i1	SIBD1_CUPSA^SIBD1_CUPSA^Q:10-291,H:11-97^42.553%ID^E:1.01e-16^RecName: Full=Single insulin-like growth factor-binding domain protein-1	.
TRINITY_DN1723_c0_g1	TRINITY_DN1723_c0_g1_i2	SIBD1_CUPSA^SIBD1_CUPSA^Q:48-344,H:1-97^46.465%ID^E:2.7e-22^RecName: Full=Single insulin-like growth factor-binding domain protein-1	SIBD1_CUPSA^SIBD1_CUPSA^Q:1-99,H:1-97^46.465%ID^E:5.02e-23^RecName: Full=Single insulin-like growth factor-binding domain protein-1
TRINITY_DN52826_c0_g1	TRINITY_DN52826_c0_g1_i1	SIBD2_CUPSA^SIBD2_CUPSA^Q:261-401,H:28-74^57.447%ID^E:1.15e-10^RecName: Full=Single insulin-like growth factor-binding domain protein-2	SIBD2_CUPSA^SIBD2_CUPSA^Q:39-115,H:24-97^41.558%ID^E:1.53e-11^RecName: Full=Single insulin-like growth factor-binding domain protein-2
TRINITY_DN1379_c0_g1	TRINITY_DN1379_c0_g1_i1	TXC20_CUPSA^TXC20_CUPSA^Q:78-263,H:3-65^52.381%ID^E:1.23e-14^RecName: Full=Toxin CSTX-20	TXC20_CUPSA^TXC20_CUPSA^Q:26-104,H:3-84^52.439%ID^E:1.9e-19^RecName: Full=Toxin CSTX-20
TRINITY_DN1379_c0_g1	TRINITY_DN1379_c0_g1_i1	TXC20_CUPSA^TXC20_CUPSA^Q:78-263,H:3-65^52.381%ID^E:1.23e-14^RecName: Full=Toxin CSTX-20	.
TRINITY_DN1379_c0_g1	TRINITY_DN1379_c0_g1_i2	TXC20_CUPSA^TXC20_CUPSA^Q:78-263,H:3-65^52.381%ID^E:2.34e-14^RecName: Full=Toxin CSTX-20	.
TRINITY_DN1379_c0_g1	TRINITY_DN1379_c0_g1_i2	TXC20_CUPSA^TXC20_CUPSA^Q:78-263,H:3-65^52.381%ID^E:2.34e-14^RecName: Full=Toxin CSTX-20	TXC20_CUPSA^TXC20_CUPSA^Q:26-104,H:3-84^52.439%ID^E:1.9e-19^RecName: Full=Toxin CSTX-20
TRINITY_DN1645_c0_g1	TRINITY_DN1645_c0_g1_i1	TXC20_CUPSA^TXC20_CUPSA^Q:249-491,H:1-82^30.488%ID^E:7.62e-07^RecName: Full=Toxin CSTX-20	TXC20_CUPSA^TXC20_CUPSA^Q:20-100,H:1-82^30.488%ID^E:1.11e-07^RecName: Full=Toxin CSTX-20
TRINITY_DN1645_c0_g1	TRINITY_DN1645_c0_g1_i2	TXC20_CUPSA^TXC20_CUPSA^Q:276-518,H:1-82^30.488%ID^E:1.06e-06^RecName: Full=Toxin CSTX-20	TXC20_CUPSA^TXC20_CUPSA^Q:29-109,H:1-82^30.488%ID^E:1.89e-07^RecName: Full=Toxin CSTX-20
