# Supplementary material for: Transcriptome analysis of the spider Phonotimpus pennimani reveals novel toxin transcripts
Source: J Venom Anim Toxins Incl Trop Dis. 2023 Jan 23;29:e20220031. doi: 10.1590/1678-9199-JVATITD-2022-0031 (PMC9881743; doi:10.1590/1678-9199-JVATITD-2022-0031)
Supplement: Additional file 3. [file 1678-9199-jvatitd-29-e20220031-s3.pdf]

J Venom Anim Toxins incl Trop Dis
https://doi.org/10.1590/1678-9199-JVATITD-2022-0031

Supplementary Material to "Transcriptome analysis of the spider *Phonotimpus pennimani* reveals novel toxin transcripts"

Additional file 3. Toxins and toxin-like peptides from Arachnida that show similarity to amino acid sequences derived from the *Phonotimpus pennimani* transcriptome.

Species	Family	Toxin name	Toxin information	ID UniProtKB	References AF
Loxosceles hirsuta	Sicariidae	Dermonecrotic toxin LhSicTox-alphaIV1i	Dermonecrotic toxins cleave the phosphodiester bond between the phosphate and headgroup of certain phospholipids (sphingolipid and lysolipid substrates), yielding an alcohol (usually choline) and a cyclic phosphate.	A411_LOXHI	Binford *et al.* [1]
Lychas mucronatus	Buthidae	Venom protein 164	AVIT protein family (prokineticin), potent prokineticin receptor agonists that induce smooth muscle contractions and hyperalgesia.	VP164_LYCMC	Ruiming *et al.* [2]
Loxosceles intermedia	Sicariidae	Dermonecrotic toxin LiSicTox-betaID1	Acts on sphingomyelin.	B1Q_LOXIN	da Silveira *et al.* [3]
Loxosceles intermedia	Sicariidae	Toxin 1 of metalloproteinase similar to astacin	Its role in venom is not completely understood, but possibly acts as a spreading factor that facilitates the diffusion of other venom components. Alternatively, it may be involved in the proteolytic action of other toxins.	VMPA_LOXIN	da Silveira *et al.* [3]
Lycosa singoriensis	Lycosidae	Structure similar to toxin LSTX-D1	Structurally similar to toxin LSTX-D1	TXZ01_LYCSI	Zhang *et al.* [4]
Lycosa singoriensis	Lycosidae	U5-lycotoxin-Ls1a	Structurally similar to toxin LSTX-E1	TX501_LYCSI	Zhang *et al.* [4]
Lycosa singoriensis	Lycosidae	U6-lycotoxin-Ls1f	Structurally similar to toxin LSTX-F6	TX606_LYCSI	Zhang *et al.* [4]
Lycosa singoriensis	Lycosidae	U6-lycotoxin-Ls1b	Structurally similar to LSTX-F10; belongs to the calcium channel	TX610_LYCSI	Zhang *et al.* [4]

			inhabiting CSTX neurotoxin family		
Lycosa singoriensis	Lycosidae	U14-lycotoxin-Ls1b	Structurally similar to toxin LSTX-N4	TXE04_LYCSI	Zhang *et al.* [4]
Agelena orientalis	Agelenidae	U7-agatoxin-Ao1a	Family member of protein 11 from venom 02 (wap-2).	TXAG7_AGEOR	Kozlov *et al.* [5]
Agelena orientalis	Agelenidae	U8-agatoxin-Ao1a	Member of the neurotoxic plectoxin family	TXAG8_AGEOR	Kozlov *et al.* [5]
Caerostris extrusa	Araneidae	U3-aranetoxin-Ce1a	Neurotoxic peptide	TXCA_CAEEX	Dai *et al.* [6]
Loxosceles spinulosa	Sicariidae	Dermonecrotic toxin LspiSicTox-betaIE1ii	Acts on sphingomyelin.	B1R2_LOXSN	Binford *et al.* [1]
Lycosa singoriensis	Lycosidae	U7-lycotoxin-Ls1c	Structurally similar to toxin LSTX-G11	TX711_LYCSI	Zhang *et al.* [4]
Lycosa singoriensis	Lycosidae	U6-lycotoxin-Ls1c	Structurally similar to toxin LSTX-F2	TX602_LYCSI	Zhang *et al.* [4]
Lycosa singoriensis	Lycosidae	U9-lycotoxin-Ls1a	Structurally similar to toxin LSTX-H30	TX830_LYCSI	Zhang *et al.* [4]
Ixodes scapularis	Ixodidae	Dermonecrotic toxin SPH	Acts on sphingomyelin.	SMD_IXOSC	Alarcon-Chaidez *et al.* [7]
Lycosa singoriensis	Lycosidae	U20-lycotoxin-Ls1d	Structurally similar to toxin LSTX-Q4; exhibits antibacterial activity	TXK04_LYCSI	Zhang *et al.* [4]
Agelena orientalis	Agelenidae	U4-agatoxin-Ao1a		TXAG4_AGEOR	Kozlov *et al.* [5]
Loxosceles intermedia	Sicariidae	Toxin 2 of metalloproteinase similar to astacin	Zinc metalloproteinase. Possibly acts as a spreading factor that facilitates the diffusion of other venom components. Alternatively, it may be involved in extraoral prey digestion or the proteolytic action of other toxins.	VMPA2_LOXIN	Trevisan-Silva *et al.* [8]
Phoneutria nigriventer	Ctenidae	U24-ctenitoxin-Pn1a	Cysteine proteinase inhibitor	PN16_PHONI	Richardson *et al.* [9]
Phoneutria nigriventer	Ctenidae	Kappa-ctenitoxin-Pn1a	Antagonist of L-type calcium channel (Cav1/CACNA1)	TX31_PHONI	Kushmerick *et al.* [10]
Phoneutria nigriventer	Ctenidae	U6-ctenitoxin-Pn1a	Antagonist of L-type calcium channel (Cav1/CACNA1)	TX3A_PHONI	Kalapothakis *et al.* [11]

Cupiennius salei	Ctenidae	Toxin CSTX-20	Neurotoxin family member 20	TXC20_CUPSA	Trachsel *et al.* [12]
Phoneutria nigriventer	Ctenidae	U21-ctenitoxin-Pn1a	Hydrolyses gelatine and succinyl casein	PN47_PHONI	Richardson *et al.* [9]

References to Additional file 3

1. Binford GJ, Bodner MR, Cordes MH, Baldwin KL, Rynerson MR, Burns SN, Zobel-Thropp PA. Molecular evolution, functional variation, and proposed nomenclature of the gene family that includes sphingomyelinase D in sicariid spider venoms. Mol Biol Evol. 2009 Mar;26(3):547-66. doi: 10.1093/molbev/msn274. Epub 2008 Nov 28.

2. Ruiming Z, Yibao M, Yawen H, Zhiyong D, Yingliang W, Zhijian C, Wenxin L. Comparative venom gland transcriptome analysis of the scorpion *Lychas mucronatus* reveals intraspecific toxic gene diversity and new venomous components. BMC Genomics. 2010 July;11:452. doi: 10.1186/1471-2164-11-452.

3. da Silveira RB, Wille AC, Chaim OM, Appel MH, Silva DT, Franco CR, Toma L, Mangili OC, Gremski W, Dietrich CP, Nader HB, Veiga SS. Identification, cloning, expression and functional characterization of an astacin-like metalloprotease toxin from *Loxosceles intermedia* (brown spider) venom. Biochem J. 2007 Sep 1;406(2):355-63. doi: 10.1042/BJ20070363.

4. Zhang Y, Chen J, Tang X, Wang F, Jiang L, Xiong X, Wang M, Rong M, Liu Z, Liang S. Transcriptome analysis of the venom glands of the Chinese wolf spider *Lycosa singoriensis*. Zoology (Jena). 2010 Jan;113(1):10-8. doi: 10.1016/j.zool.2009.04.001.

5. Kozlov S, Malyavka A, McCutchen B, Lu A, Schepers E, Herrmann R, Grishin E. A novel strategy for the identification of toxinlike structures in spider venom. Proteins. 2005 Apr 1;59(1):131-40. doi: 10.1002/prot.20390.

6. Dai L, Yasuda A, Naoki H, Corzo G, Andriantsiferana M, Nakajima T. IsCT, a novel cytotoxic linear peptide from scorpion *Opisthacanthus madagascariensis*. Biochem Biophys Res Commun. 2001 Aug 31;286(4):820-5. doi: 10.1006/bbrc.2001.5472.

7. Alarcon-Chaidez FJ, Sun J, Wikel SK. Transcriptome analysis of the salivary glands of *Dermacentor andersoni* Stiles (Acari: Ixodidae). Insect Biochem Mol Biol. 2007 Jan;37(1):48-71. doi: 10.1016/j.ibmb.2006.10.002.

8. Trevisan-Silva D, Gremski LH, Chaim OM, da Silveira RB, Meissner GO, Mangili OC, Barbaro KC, Gremski W, Veiga SS, Senff-Ribeiro A. Astacin-like metalloproteases are a gene family of toxins present in the venom of different species of the brown spider (genus *Loxosceles*). Biochimie. 2010 Jan;92(1):21-32. doi: 10.1016/j.biochi.2009.10.003.

9. Richardson M, Pimenta AMC, Bemquerer MP, Santoro MM, Beirao PSL, Lima ME, Figueiredo SG, Bloch C Jr, Vasconcelos EAR, Campos FAP, Gomes PC, Cordeiro MN. Comparison of the partial proteomes of the venoms of Brazilian spiders of the genus *Phoneutria*. Comp Biochem Physiol C Toxicol Pharmacol. 2006 Mar-Apr;142(3-4):173-187. doi: 10.1016/j.cbpc.2005.09.010.

10. Kushmerick C, Kalapothakis E, Beirão PS, Penaforte CL, Prado VF, Cruz JS, Diniz CR, Cordeiro MN, Gomez MV, Romano-Silva MA, Prado MA. *Phoneutria nigriventer* toxin Tx3-1 blocks A-type K+ currents controlling Ca2+ oscillation frequency in GH3 cells. J Neurochem. 1999 Apr;72(4):1472-81. doi: 10.1046/j.1471-4159.1999.721472.x.

11. Kalapothakis E, Penaforte CL, Leão RM, Cruz JS, Prado VF, Cordeiro MN, Diniz CR, Romano-Silva MA, Prado MA, Gomez MV, Beirão PS. Cloning, cDNA sequence analysis and patch clamp studies of a toxin from the venom of the armed spider (*Phoneutria nigriventer*). Toxicon. 1998 Dec;36(12):1971-80. doi: 10.1016/s0041-0101(98)00127-5.

12. Trachsel C, Siegemund D, Kämpfer U, Kopp LS, Bühr C, Grossmann J, Lüthi C, Cunningham M, Nentwig W, Kuhn-Nentwig L, Schürch S, Schaller J. Multicomponent venom of the spider *Cupiennius salei*: a bioanalytical investigation applying different strategies. FEBS J. 2012 Aug;279(15):2683-94. doi: 10.1111/j.1742-4658.2012.08650.x.
