# Supplementary material for: Transcriptome analysis of the spider Phonotimpus pennimani reveals novel toxin transcripts
Source: J Venom Anim Toxins Incl Trop Dis. 2023 Jan 23;29:e20220031. doi: 10.1590/1678-9199-JVATITD-2022-0031 (PMC9881743; doi:10.1590/1678-9199-JVATITD-2022-0031)
Supplement: Additional file 4. [file 1678-9199-jvatitd-29-e20220031-s4.pdf]

J Venom Anim Toxins incl Trop Dis
https://doi.org/10.1590/1678-9199-JVATITD-2022-0031

Supplementary Material to "Transcriptome analysis of the spider *Phonotimpus pennimani* reveals novel toxin transcripts"

Additional file 4. Elongation factor-1 alpha (EloFa) and succinate dehydrogenase (SD) have been used as RT-qPCR reference in various developmental stages of spider mites [54]. According to software analysis of candidate reference genes (GeNorm, NormFinder, and Bestkeeper). EloFa and SD genes are stable under stress conditions. We submitted our qPCR data to the same software analysis and also to the comparative delta-Ct method through the web-based tool RefFinder [57]. Results from the four software analyses are shown in **(A)** ranking order, better-good-average; and **(B)** comprehensive ranking, Geomean values: SD or SuccD = 1.19 and EloFa = 1.41, considering that lower ranking values indicate higher gene stability.

(A) Ranking order (Better–Good–Average)

Method	Gene 1 (Better)	Gene 2 (Good)
Delta CT	EloFa	SD
BestKeeper	SD	EloFa
Normfinder	SD	EloFa
Genorm	EloFa	SD

(B) Comprehensive ranking

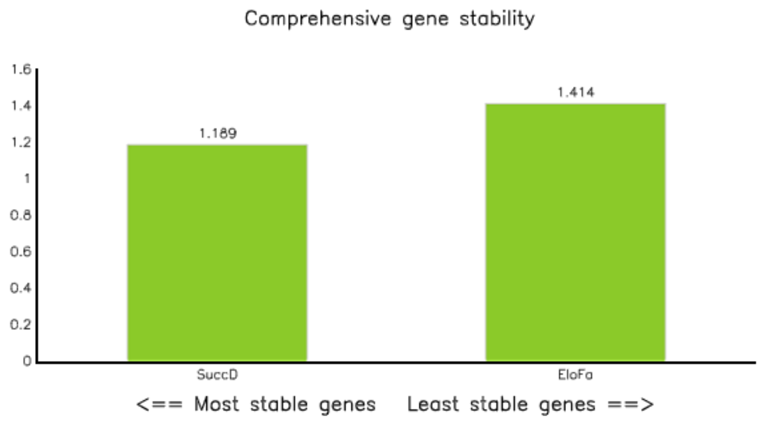

References to Additional file 1

1. **BestKeeper**: Pfaffl MW, Tichopad A, Prgomet C, Neuvians TP. Determination of stable housekeeping genes, differentially regulated target genes and sample integrity: BestKeeper--Excel-based tool using pair-wise correlations. Biotechnol Lett. 2004 Mar;26(6):509-15. doi: 10.1023/b:bile.0000019559.84305.47.

2. **NormFinder**: Andersen CL, Jensen JL, Ørntoft TF. Normalization of real-time quantitative reverse transcription-PCR data: a model-based variance estimation approach to identify genes suited for normalization, applied to bladder and colon cancer data sets. Cancer Res. 2004 Aug 1;64(15):5245-50. doi: 10.1158/0008-5472.CAN-04-0496.
3. **Genorm**: Vandesompele J, De Preter K, Pattyn F, Poppe B, Van Roy N, De Paepe A, Speleman F. Accurate normalization of real-time quantitative RT-PCR data by geometric averaging of multiple internal control genes. Genome Biol. 2002 Jun 18;3(7):RESEARCH0034. doi: 10.1186/gb-2002-3-7-research0034. Epub 2002 Jun 18.
4. **The comparative delta-Ct method**: Silver N, Best S, Jiang J, Thein SL. Selection of housekeeping genes for gene expression studies in human reticulocytes using real-time PCR. BMC Mol Biol. 2006 Oct 6;7:33. doi: 10.1186/1471-2199-7-33.
