# Supplementary material for: Transcriptome analysis of the spider Phonotimpus pennimani reveals novel toxin transcripts
Source: J Venom Anim Toxins Incl Trop Dis. 2023 Jan 23;29:e20220031. doi: 10.1590/1678-9199-JVATITD-2022-0031 (PMC9881743; doi:10.1590/1678-9199-JVATITD-2022-0031)

J Venom Anim Toxins incl Trop Dis
https://doi.org/10.1590/1678-9199-JVATITD-2022-0031

Supplementary Material to "Transcriptome analysis of the spider *Phonotimpus pennimani* reveals novel toxin transcripts"

Additional file 5. Primers were validated through melting curves to ensure a single PCR product. After each RT-PCR run, amplicons were visualized using 2% agarose gels, as can be seen in the following images. About 10 µL PCR amplicon was loaded in 2% agarose gel. **(A)** ICK motif that corresponds to CIP-stop fragment; **(B)** complete PpenTox1 or Met-stop fragment; **(C)** and **(D)** elongation factor-1-alpha and succinate dehydrogenase fragments. L: 50 bp DNA ladder; Ju: population of juveniles; F: females; M: males; and NTC: not template curves.

(A) PhoTox1CIP/PhoTox1STOP fragment (amplicon 201 bp; primer 2 and 3)

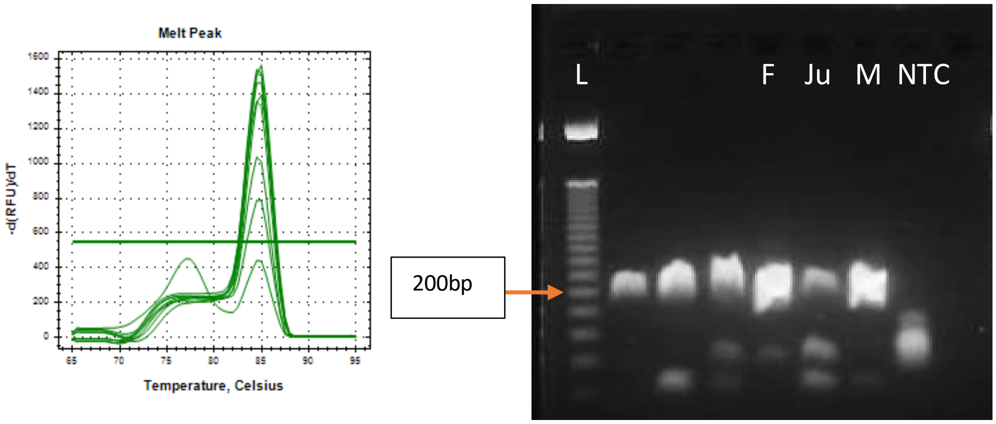

(B) PhoTox1MET/PhoTox1STOP fragment (amplicon 381 bp; primer 1 and 3)

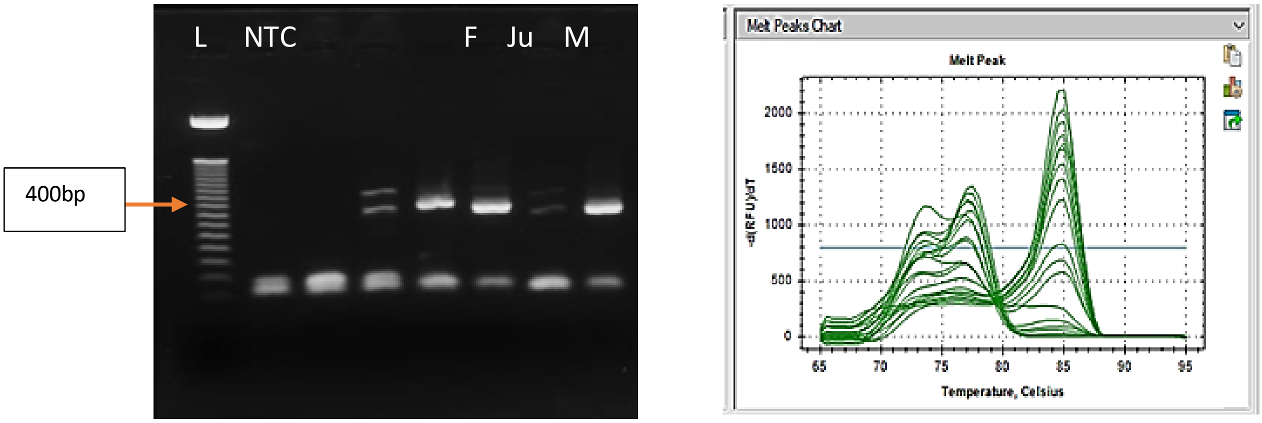

(C) EloFa fragment (EloFa_For/EloFa_Rev primers, amplicon 218 bp)

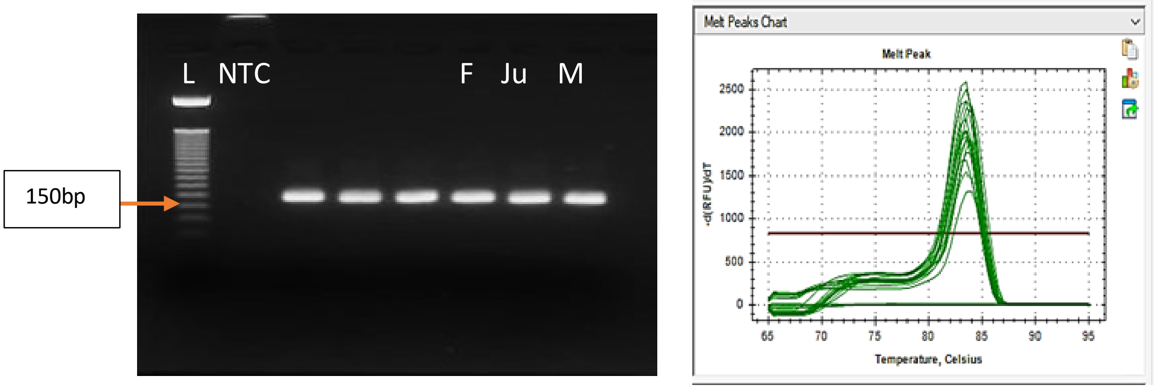

(D) SD (SuccDe_For/SuccDe_Rev primers, amplicon 137 bp)

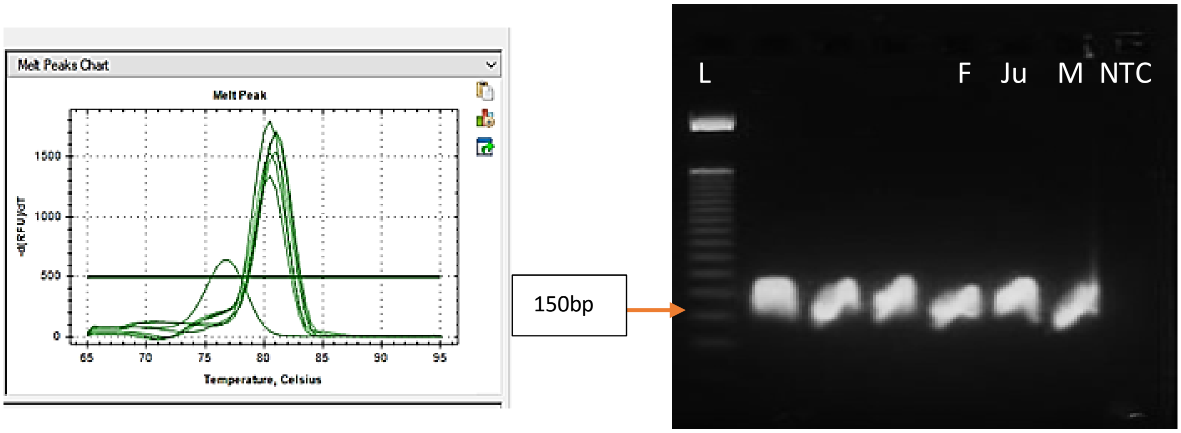

Supplement: Additional file 5. [file 1678-9199-jvatitd-29-e20220031-s5.pdf]
